# Steelhead (*Oncorhynchus mykiss*) lineages and sexes show variable patterns of association of adult migration timing and age‐at‐maturity traits with two genomic regions

**DOI:** 10.1111/eva.13088

**Published:** 2020-08-27

**Authors:** Stuart C. Willis, Jon E. Hess, Jeff K. Fryer, John M. Whiteaker, Chris Brun, Ryan Gerstenberger, Shawn R. Narum

**Affiliations:** ^1^ Hagerman Genetics Laboratory Columbia River Inter‐Tribal Fish Commission Hagerman ID USA; ^2^ Fishery Science Department Columbia River Inter‐Tribal Fish Commission Portland OR USA; ^3^ Branch of Natural Resources – Fisheries, Confederated Tribes of Warm Springs Portland OR USA

**Keywords:** genome‐wide association study, genotype–phenotype association, haplotype, rainbow trout, salmon

## Abstract

As life history diversity plays a critical role in supporting the resilience of exploited populations, understanding the genetic basis of those life history variations is important for conservation management. However, effective application requires a robust understanding of the strength and universality of genetic associations. Here, we examine genetic variation of single nucleotide polymorphisms in genomic regions previously associated with migration phenology and age‐at‐maturity in steelhead (*Oncorhynchus mykiss*) from the Columbia River. We found chromosome 28 markers (GREB1L, ROCK1 genes) explained significant variance in migration timing in both coastal and inland steelhead. However, strength of association was much greater in coastal than inland steelhead (*R* ^2^ 0.51 vs. 0.08), suggesting that genomic background and challenging inland migration pathways may act to moderate effects of this region. Further, we found that chromosome 25 candidate markers (SIX6 gene) were significantly associated with age and size at first return migration for inland steelhead, and this pattern was mediated by sex in a predictable pattern (males *R* ^2^ = 0.139–0.170; females *R* ^2^ = 0.096–0.111). While this encourages using these candidate regions in predicting life history characteristics, we suggest that stock‐specific associations and haplotype frequencies will be useful in guiding implementation of genetic assays to inform management.

## 1 INTRODUCTION

For protected species under management, ensuring that genetic and phenotypic diversity is maintained above critical levels is one of the primary goals of conservation (Funk, McKay, Hohenlohe, & Allendorf, 2012; Hoelzel, Bruford, & Fleischer, 2019; Waples & Lindley, 2018). Intraspecific phenotypic diversity including trophic and life history variation is critical for preserving the resilience of populations to short term environmental fluctuations, as well as ensuring their ability to adapt to long‐term environmental or anthropogenic challenges (Hoelzel et al., 2019). This diversity also increases stability in ecosystem roles and services in which the species participates (i.e., portfolio effects; Moore, Yeakel, Peard, Lough, & Beere, 2014; Schindler, Armstrong, & Reed, 2015; Schindler et al., 2010).

Genetic diversity often serves as an important proxy for unmeasured contemporary phenotypic diversity or adaptive capacity from standing genetic variation (Funk et al., 2012; Hoelzel et al., 2019; Waples & Lindley, 2018). However, in many cases prominent phenotypic characteristics are well known to managers since they can serve as indicators for important applications such as mixed stock analyses or detection of differential pressure from anthropogenic activities (Hare & Richardson, 2013; Trippel, 1995). Nonetheless, the reliability of these phenotypes to serve as indicators of demographic trends depends on the ability of managers to accurately identify and measure these traits. For traits with heritable components, the ability to survey allelic variants strongly associated with phenotypic traits may provide managers with a more direct way to assess the phenotypic portfolio of a population and measure changes across space and time (Moran, Bromaghin, & Masuda, 2014). Moreover, with the expansion of next‐generation sequencing protocols and platforms, the ability to discover and survey markers associated with important phenotypic variants has greatly increased (Ouborg, Pertoldi, Loeschcke, Bijlsma, & Hedrick, 2010). However, numerous questions remain regarding the efficacy of applying genetic predictions of phenotypic variants, or their ecological and evolutionary implications, in the context of population management (Kardos & Shafer, 2018; Pearse, 2016; Waples & Lindley, 2018).

In Pacific salmon listed under the Endangered Species Act (ESA), adult migration timing and age‐at‐maturity (age at first return migration) are two key traits that are commonly used in conservation management (National Marine Fisheries Service, 2020). Life history variation differs widely among salmonids, but rainbow trout (*Oncorhynchus mykiss*), the anadromous forms of which are called steelhead, is recognized as a species that displays broad phenotypic diversity for these two traits, each of which is known to be under strong genetic influence (Carlson & Seamons, 2008).

### 1.1 Migration timing

It has long been observed that there is multi‐modality in migration return, or multiple “runs”, of steelhead throughout the year (Busby, Wainwright, & Bryant, 1996). In effect, these consist of a portion of the population that begins their migration to spawning grounds early, in a reproductively premature state, and hold in or near spawning grounds for some weeks or months before spawning, while another portion returns later, having matured in the ocean, and arrive immediately prior to spawning (Busby et al., 1996; Quinn, McGinnity, & Reed, 2016). In Columbia River steelhead, these premature or “summer‐run” fish overwinter in freshwater in contrast to mature or “winter‐run” fish that spawn soon after arriving (Busby et al., 1996; Quinn et al., 2016). Both runs spawn in the spring with different but overlapping timing (Quinn et al., 2016), such that fish of different run types that migrate to the same stream are usually more genetically similar than to those of the same run type in other tributaries (Prince et al., 2017; Waples & Lindley, 2018 and references therein).

Although it has been known for some time that run timing is strongly heritable (Carlson & Seamons, 2008; Quinn, Unwin, & Kinnison, 2000), only recently was a genomic region of large effect discovered by comparing the variation in reduced representation genomic sequencing of individuals from each run type returning to the same tributary (Hess, Zendt, Matala, & Narum, 2016; Prince et al., 2017), and was mapped more finely through whole‐genome resequencing (Micheletti, Hess, Zendt, & Narum, 2018) in coastal steelhead populations from California, Oregon, and the lower Columbia basin. This region, located on chromosome 28, contains two genes, the human homologs for which are “growth regulation by estrogen in breast cancer gene like” or “*GREB1* Like retinoic acid receptor coactivator” (*GREB1L*) and “rho‐associated coiled‐coil containing protein kinase 1” (*ROCK1*) (Hess, Zendt, et al., 2016; Micheletti, Hess, et al., 2018). Both genes are understood to be involved in pathways affecting gonadal development, actin‐myosin contraction, and renal development, as well as expression in renal and reproductive tissues (Brophy et al., 2017; De Tomasi et al., 2017; Mizuno et al., 2013; Nakagawa et al., 1996; Oviedo et al., 2011; Sanna‐Cherchi et al., 2017), and so could credibly be involved in reproductive maturation and environmental acclimation in anadromous salmonids. However, it remains unclear which of these genes, or their upstream regulatory regions, is most strongly associated with maturation and run timing, and if these markers are effective for predicting run timing in any given population or stock (Waples & Lindley, 2018).

**Figure 1:**
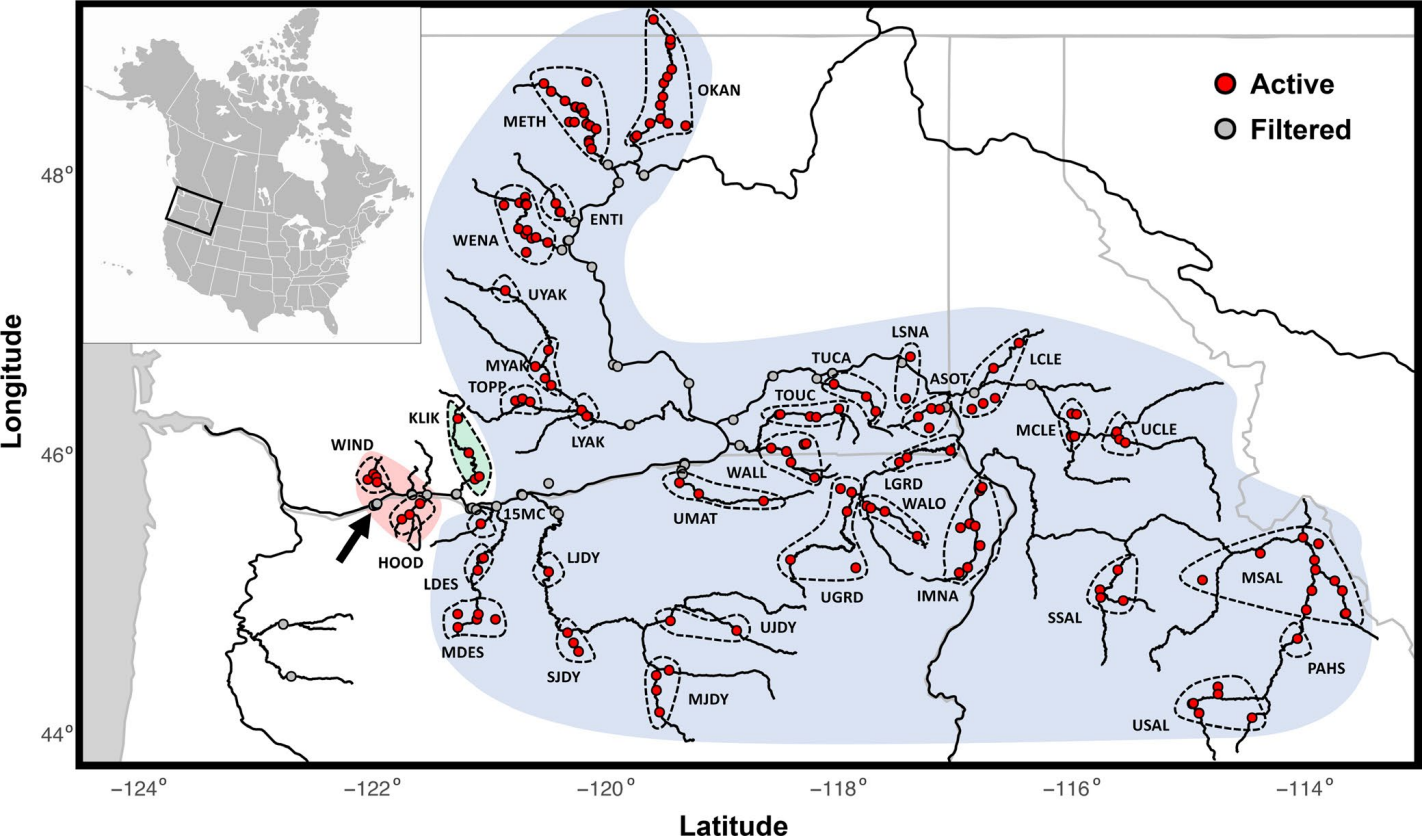
Columbia River Basin with active and filtered passive integrated transponder arrays. River courses in black; borders and coast in gray. Hydrological unit groupings, identified by dashed lines, are organized into lineages and sub‐basins as follows: coastal lineage in Red: Hood (HOOD); Wind (WIND); intermediate lineage affiliation in Green: Klickitat (KLIK); inland lineage in Blue: Fifteen Mile Creek (15MC); Deschutes (LDES, MDES); John Day (LJDAY, SJDAY, MJDY, UJDAY); Umatilla (UMAT); Walla Walla (WALL, TOUC); Tucannon (TUCA); Lower Snake (LSNA, ASOT); Grande Ronde (LGRD, WALO, UGRD); Imnaha (IMNA); Clearwater (LCLE, MCLE, UCLE); Salmon (SSAL, MSAL, PASH, USAL); Yakima (LYAK, TOPP, MYAK, UYAK); Wenatchee (WENA); Entiat (ENTI); Methow (METH); Okanagan (OKAN). Bonneville Dam is identified by an arrow

There is also uncertainty to what extent chromosome 28 variation predicts migration phenology or run timing variation for populations from the two distinct lineages of steelhead in the Columbia River Basin (Figure 1; Busby et al., 1996). These two lineages are known as coastal and inland and are often considered distinct subspecies (*O. mykiss irideus* and *O. mykiss gairdneri*, respectively; Behnke, 1992), with divergence in allozymes on the order of 7% (Reisenbichler, McIntyre, Solazzi, & Landino, 1992) and high divergence between lineages shown with both microsatellites (Blankenship et al., 2011) and SNPs (Micheletti, Matala, Matala, & Narum, 2018). For coastal lineage steelhead in lower Columbia tributaries, there may often be a strong correlation between when fish enter freshwater (summer‐run vs. winter‐run), their maturation state (stream vs. ocean maturing), and when they arrive on the spawning grounds (early vs. late spring). For steelhead migrating to the interior Columbia Basin, however, several distinguishable traits may be subsumed within “run timing,” which may correlate incompletely with maturation state at the initiation or completion of migration. For example, inland lineage fish enter freshwater during summer or fall in a premature state (Quinn et al., 2016), but they exhibit a range of arrival times to spawning tributaries, where arrival time is directly influenced by whether they arrive early to overwinter in the spawning tributary or arrive later after overwintering in a higher order river (Keefer, Boggs, Peery, & Caudill, 2008). Moreover, although steelhead of the inland lineage exclusively migrate to freshwater as stream maturing fish, they do not have a high frequency of the premature‐associated alleles as might be expected (Collins, Hargrove, Delomas, & Narum, 2020; Micheletti, Hess, et al., 2018). Nevertheless, migration data for inland steelhead suggest there is an association with chromosome 28 alleles and variation in timing of arrival to spawning grounds (Micheletti, Hess, et al., 2018). Thus, time entering freshwater, time arriving on spawning grounds, and maturation state at each of those times may be separate traits with related or distinct genetic derivations, gene‐by‐environment interactions, and dependence on genomic background.

### 1.2 Age‐at‐maturity

Steelhead vary in the amount of time they rear in freshwater before they smoltify and migrate to the sea, as well as the amount of time that they spend in the ocean before returning to freshwater to spawn, and thus exhibit a wide range of ages at first reproduction, hereafter “age‐at‐maturity” (Brannon, Powell, Quinn, & Talbot, 2004). However, as growth rates are much higher in the sea than rivers, there is a strong correlation between body size (i.e., fork length) and the “ocean‐age” of the fish (Copeland, Ackerman, Wright, & Byrne, 2017). This variation in age at first return migration, commonly known as age‐at‐maturity, along with iteroparous spawning, provides greater overlap among generations and buffers the population against poor returns for individual year classes and increases genetic diversity (Bisson, Dunham, & Reeves, 2009; Moore et al., 2014). Steelhead in the Columbia River typically spend 1 or 2 years in the ocean, with more rare occurrences of fish that spend 3 or 4 years in salt water (Busby et al., 1996). While most populations exhibit variation in age‐at‐maturity, certain Snake River tributary populations have historically been predominately 2+‐ocean fish that achieve large body size (Bowersox, Corsi, McCormick, Copeland, & Campbell, 2019; Copeland et al., 2017). However, as with run timing, geographically proximal populations that differ in predominant age‐at‐maturity are nonetheless more genetically similar than more distant populations of the same predominant age (Hess, Ackerman, et al., 2016). Although genetic stock identification is used to complement fisheries management objectives, categories of fork lengths are used as proxies for ESA‐listed Snake River populations to manage steelhead fisheries in the Columbia Basin (Copeland et al., 2017; Keefer et al., 2018). Given that length is an imperfect proxy for identifying the biological mechanism that mediates size and age in steelhead in the Columbia Basin, predicting age‐at‐maturity using genetic data would be a useful tool.

Strong heritability of age‐at‐maturity in salmonids has been known for some time, including in steelhead (Busby et al., 1996; Tipping, 1991), although only recently have some genetic underpinnings of this trait come to light. In a SNP array and whole‐genome resequencing study of European Atlantic salmon (*Salmo salar*), two genes, whose human homologs are known as “vestigial like family member 3” (*VGLL3*) and “sine oculis homeobox homolog 6” (*SIX6*), showed strong associations with ocean‐age (Barson et al., 2015; Sinclair‐Waters et al., 2020). In contrast, a recent study of steelhead using whole‐genome resequencing data found a consistent signal of association only in the region of chromosome 25 containing the *SIX6* gene among populations in the interior Columbia basin, Puget Sound (WA), and California, and developed several candidate genomic markers for age‐at‐maturity (C.D. Waters et al., 2020). This gene, which is known in humans to have effects on age‐at‐menarche as well as adult height (Perry et al., 2014), may influence age‐at‐maturity through adipogenesis and gametogenesis (Jean, Bernier, & Gruss, 1999; Kurko et al., 2019; Larder, Clark, Miller, & Mellon, 2011). While an apparent candidate gene for age‐at‐maturity in steelhead, it remains unclear whether markers in this region are predictive of the trait and how strong the correlation remains across populations.

### 1.3 Study objectives

While the application of new genetic data to population management may seem obvious, numerous uncertainties about the patterns of association of these genomic regions and life history variation need to be resolved before widespread application in conservation management (Pearse, 2016; Waples & Lindley, 2018). Among these, important knowledge gaps include the following: (a) the nature and strength of association in regional populations with unique recombinant frequencies and genomic backgrounds, (b) the predictive value of different genetic markers for life history traits in those populations, and (c) the frequency and trait values of heterozygotes. In this study, we address these uncertainties to clarify the utility of applying candidate markers for conservation management for steelhead in Columbia River.

## 2 METHODS

### 2.1 Samples

Our data derive from two groups of steelhead (*Oncorhynchus mykiss*) samples from the Columbia River basin. Upon entering the Columbia River basin, adult fish migrating to the Columbia interior (east of the Cascades Range) first encounter Bonneville Dam, and the date of this arrival served as an approximate estimate of the date of freshwater entry. A portion of adult steelhead were sampled (~2% of total run; Hess, Ackerman, et al., 2016) as they ascended the fish ladder at Bonneville Dam Adult Fish Facility (BONAFF) from April through October each year, and using procedures approved by the Fish Passage Operation and Maintenance Coordination Team. We note that sampling at Bonneville Dam was reduced when water temperatures exceeded 21.1°C and then suspended when water temperatures exceed 22.2°C (70°F), usually for a few weeks during the peak of the run in August or September each year. For each fish, fork length was measured, a PIT tag inserted (unless previously tagged), scales taken for aging, and a fin clip taken for genetic analysis. Fish were then released to continue their migration. Our first dataset consisted of fish sampled from 2013 to 2018 (spawn years 2014–2019) that arrived at or near their spawning site as determined from PIT tag data (*N* = 1,538; other samples were rejected based on filtering described below). Data available for these fish included their fork length, river, and ocean‐ages (from scale readings), and the dates they were recorded at PIT arrays positioned throughout the Columbia River basin (Figure 1). These data were used to infer phenotypes related to migration timing and ocean‐age‐at‐maturity.

Only a few tributaries upstream of Bonneville Dam exhibit significant runs of fish that enter freshwater reproductively mature during the winter and spring (winter‐run), and few of these will have been sampled at BONAFF. Thus, our BONAFF samples largely represented fish that would be considered summer‐run from the inland lineage. However, it also includes a few summer‐run coastal lineage fish that predominantly pass through Bonneville during the Skamania Summer Steelhead Management Period (April 1–June 30; Hess, Zendt, et al., 2016), named for the predominance of fish that migrate during this time that are derived from the Skamania Hatchery stock. In order to make an adequate comparison with both winter‐run and summer‐run fish, we also assembled a dataset from the Hood River (Figure 1), which hosts solely coastal lineage fish that arrive year‐round in premature (summer‐run, stream maturing) or mature (winter‐run, ocean maturing) states. The Hood River tissue samples (*N* = 354) were taken from adult fish sampled for a hatchery broodstock program between 2007 and 2019, and so the dates they arrived in the Hood River were also archived in PIT databases. A subset of these samples (*N* = 77) were also sampled at traps as juveniles and PIT tagged during their migration to the sea, providing recordings of when they passed Bonneville during their adult return migration and making them comparable to the BONAFF samples described above. It should be noted that, because the broodstock sampling program targets Hood River winter‐run fish, samples in this dataset were biased to mature migrating fish. Importantly, as only a minor portion of the total run is sampled at Bonneville Dam, and none from November to March, the Hood River samples did not overlap with BONAFF samples, providing two datasets for association analyses that were statistically independent from each other, and from the samples from which the candidate markers were originally discovered (Wray et al., 2013).

### 2.2 Bonneville dam migration timing

For all fish sampled at Bonneville between 2013 and 2018, we obtained complete PIT histories from PTAGIS (https://www.ptagis.org), including the PIT array name, river kilometer, and date of recording (*N* = 6,794). These were imported into R (R Corp.), where they were filtered using a custom routine (File S1). For each fish, the most upstream array at which it was recorded was expected to be the closest to the tributary in which they spawned and provided a reasonable estimate of arrival date. Pre‐arrival mortalities were expected to exhibit incomplete migration histories, such as being last recorded at a mainstem site where spawning is not expected, and were filtered out of the PIT tag data. To avoid pre‐arrival mortalities and insufficient precision in arrival date, we filtered records for which the most upstream array was a mainstem dam (Keefer & Caudill, 2014; Keefer et al., 2018), as well as sites in the lower Wenatchee, Methow, Entiat, and Clearwater, since fish recorded at these arrays may be distributing to a number of different tributaries higher in each sub‐basin (Figure 1). To avoid inaccuracy introduced by “dip‐ins,” fish that temporarily seek thermal refuge before resuming migration to natal sites (Keefer, Boggs, et al., 2008), we filtered records with most upstream recordings at arrays near the mouths of the Klickitat, Hood, Deschutes or John Day Rivers, which often serve as temporary thermal shelter locations for steelhead (High, Peery, & Bennett, 2006; Keefer & Caudill, 2014). For each record passing these filters, we recorded the date this fish was first recorded at Bonneville Dam (hereafter Bonneville passage day), a proxy of migration initiation and freshwater entry, and the date the fish was recorded at the most upstream PIT array (hereafter tributary arrival day), a proxy for arrival to spawning tributary, both converted to ordinal days (sequential days beginning with January 1 of the year that fish passed Bonneville). To filter repeat migrating steelhead that were attempting iteroparous spawning, we filtered records with two upstream or Bonneville detections that were nearly a year apart and/or where the length of time between Bonneville passage day and tributary arrival day was longer than 11 months (330 days), a number determined empirically by interrogating the complete records of outliers in plots of Bonneville passage versus tributary arrival day (see also Busby et al., 1996). To avoid the mistaken inclusion of late‐arriving winter‐run fish, which may overlap with early summer‐run fish in the Hood and Wind Rivers and disrupt spawn year progression by ordinal day (see more below), we omitted records for fish with both Bonneville passage day before mid‐May and tributary arrival day before mid‐June. For each fish, we calculated two compound statistics, the lag time between Bonneville passage and tributary arrival days, as well as the migration rate given this interval and the distance between Bonneville Dam and each most upstream array.

PIT arrays were grouped by sub‐basin and hydrological unit (HUC), the latter modified from US Geological Survey HUC level 8 designations (Figure 1). Our modifications to the USGS HUC8 designations were done based on geographic proximity of arrays to maximize power (subsample size) while minimizing internal variation in environmental factors, which we expect to vary strongly with geography. For fish returning to these grouped arrays, we calculated two relative statistic sets: Each fish's Bonneville passage and tributary arrival day was transformed relative to the median or relative to the last passage or arrival day, respectively, for fish arriving to arrays grouped by HUC or grouped sub‐basin. We calculated each of these statistics (a) passage/arrival day relative to (b) median or last passage/arrival day by (c) HUC or sub‐basin) for fish migrating (d) only in a given year or across all years, for a total of 20 phenotypic response variables for migration timing (2 raw [passage/arrival], 2 compound [lag time/migration rate], 16 relative [2 phenotypes (passage/arrival) relative to 2 days (median/last) of 2 groupings (HUC/sub‐basin) across 2 time periods (each year/all years)]). We calculated these relative measures because fish from different HUC/sub‐basins or years may experience environmental conditions that mediate migration cues in as‐yet‐unpredictable ways, while within these groupings, genotype may be a stronger predictor of run timing. We classified fish as part of the coastal (Hood and Wind), intermediate (Klickitat), or inland (all others) lineages based on previously determined lineage affiliations of the stream to which they migrated (Busby et al., 1996; Collins et al., 2020; Hess, Ackerman, et al., 2016).

### 2.3 Hood River migration timing

Records of fish sampled in the Hood River were filtered similarly, except that no relative or compound measures were calculated, since the Hood River is a single HUC and sub‐basin. To filter for iteroparous fish, we omitted the Bonneville passage phenotype of fish with two or more Bonneville or upstream detections that were nearly a year apart and which had gaps of more than 300 days between Bonneville passage and tributary arrival days. However, in these cases we retained tributary arrival day, rather than filtering the whole record (File S2), retaining a single record for each individual. In addition, we made a modification to both dates for all Hood River records to align fish from the same spawn year in temporal order. Late‐arriving “winter‐run” fish and very early‐arriving “summer‐run” fish from consecutive spawn years may overlap in the Hood River in April/May of the same calendar year but not spawn together. Thus, to make ordinal dates consistent with spawn year, the *N* = 324 Hood River fish passing Bonneville before ordinal 121 (May 1) or arriving before ordinal 141 (May 21) in a given calendar year had 365 days added to these phenotype values (e.g., a fish that passed Bonneville on January 1, ordinal 1, and was recorded at a Hood River array February 1, ordinal 32, was analyzed with Bonneville passage and tributary arrival days 366 and 398, respectively, to place it after fish arriving the summer before but spawning the same spring). This has the effect of ensuring that the temporal order of genotypes for association testing is homozygous‐premature, heterozygous, and homozygous‐mature to avoid erroneous signals of overdominance. However, we note that some summer‐run fish arriving before these dates, as judged by candidate marker genotypes, may also have been inadvertently moved as well, which could diminish associations in Hood River samples (Figure S1).

### 2.4 Age and length phenotypes

We collated fork length, as well as scale‐based ocean‐age and total age (river + ocean, where available) from the records for each BONAFF fish. Steelhead passing Bonneville Dam are a mixture of wild (WOR) and hatchery‐origin (HOR) fish, and as HOR fish in the BONAFF samples are routinely identified using parentage‐based tagging (PBT), the natal spawn year, and thus age, are available for most HOR fish (HOR fish *N* = 645; see Hess, Zendt, et al., 2016). For those HOR fish for which both river and ocean‐age were available from scale data (*N* = 501), we correlated the total age estimates from scales to those calculated from parentage using the *cor.test* function in R. However, we made no effort to correct the scale‐based ocean‐ages, since this would not be possible for WOR fish and could introduce some bias by origin. Similarly, we did not use the PBT data to assign fish to stock or lineage, but only to calculate true total age. As the Hood River fish were not sampled at Bonneville Dam, age and length data were not available for these samples, and the association tests described below were only made with the BONAFF fish.

### 2.5 Genotyping

DNA was extracted from fin clips of each fish using nondenatured Chelex (Sigma‐Aldrich). A panel of 367 markers (SNPs and insertion‐deletion sites) were genotyped using GTseq, a pooled amplicon procedure using indexed samples (Campbell, Harmon, & Narum, 2015), on an Illumina Nextseq platform. The origins of these markers are diverse: The majority of markers were putatively neutral but with sufficient heterozygosity to be predictive of population origin (Campbell et al., 2012; Hess, Ackerman, et al., 2016), and included a marker from the sdY gene region with high predictive value for sex (Brunelli, Wertzler, Sundin, & Thorgaard, 2008), as well as markers with putative adaptive value that were identified as *F*_ST_ outliers from landscape analyses (Micheletti, Matala, et al., 2018). In addition, the panel included thirteen markers in the region of chromosomes 28 associated with migration timing (Micheletti, Matala, et al., 2018), with six from exonic and intronic regions of the *GREB1L* gene, six from the intergenic region, and one from an intron of the *ROCK1* gene (Table S1) (Collins et al., 2020). Finally, the panel included ten markers in the region of chromosome 25 associated with age and size in maturity (Waters et al., 2020) with three in the first transcribed region of the *SIX6* gene, six in the upstream intergenic region, and one in the downstream intergenic region (Table S1).

Genotypes from the GTseq pipeline were converted to various formats using PGDspider and custom code in R (R Corp.) (available at https://github.com/stuartwillis/Progeny_convert_haplotype_genotype). Data were filtered to retain samples with <10% missing data. To obtain an independent, neutral set of loci from which to infer population structure, putative neutral loci were filtered for linkage in PLINK 1.9 (Purcell et al., 2007) by sets of 50 loci in steps of 5 loci using a maximum *r* ^2^ of 0.9. These linkage‐pruned loci were then tested with Bayescan 2.1 (Foll, 2008) with default parameters except for a thinning interval of 100, and notably, a prior odds of 10. Increased prior odds (100) produced fewer outlier loci (not shown), but as we wished to retain only loci with no evidence of selection, we used the lower value of 10. We ran the Bayescan analysis twice to confirm convergence. Only loci with *q*‐values >0.1 were retained and will be referred to as the “neutral” set for subsequent analyses.

We examined whether SNPs within each of the candidate regions of chromosomes 28 and 25 were in strong linkage disequilibrium (LD), using the program Haploview v4.2 (Barrett, Fry, Maller, & Daly, 2005) to visualize pairwise *r* ^2^ for the complete BONAFF dataset as well as each lineage in the BONAFF data, and for chromosome 28 only in the Hood River samples. As a comparison to inputs for subsequent haplotype‐based analyses, we used Haploview to infer and estimate the frequency of haplotypes for each set after manually designating each contiguous region as a single haplotype block. In Haploview, haplotypes are estimated using an accelerated expectation–maximization (EM) algorithm (Qin et al. 2002, Am J Hum Genet.). Each formal SNP identifier includes their chromosome position, but for simplicity we refer to the SNP markers in each candidate region of chromosomes 28 and 25 by their order of sequence along the chromosome: one through thirteen or ten, respectively (Table S1).

### 2.6 Genetic association analyses

We tested the association of run timing statistics, as well as fork length, ocean‐age, and total age, with each of the GTseq markers except three species delimitation markers and the sex marker, in GAPIT v. 20190926 (Lipka et al., 2012). We used the 230 “neutral” loci to infer a kinship matrix and three principal components reflecting underlying population structure, with the three components explaining 2.6%, 1.7%, and 1.2% of the genetic variance in the “neutral” loci, respectively. In GAPIT, the kinship matrix is utilized as a random factor, and the principal components, along with any other covariates, are incorporated as fixed factors, in several available models. We ran two of the available GAPIT models with each of the datasets, the “mixed linear model” (MLM), which tests each SNP independently, and the “Bayesian‐information and Linkage‐disequilibrium Iteratively Nested Keyway” (BLINK) model, which first groups loci according to linkage thresholds and reports a representative SNP from each tested LD group, testing the significance of subsequent clusters while including significant LD groups as covariates (Files [Link], [Link], [Link]). We modified the BLINK code, which has a native LD threshold (*r* ^2^) of 0.7, to group only loci in near‐perfect LD (≥0.999), implying that differences in significance between the MLM and BLINK models reflects the redundancy in explanatory power produced by the remaining linkage among loci. The BLINK model does not report the variance explained by each LD group (*R* ^2^), so for the first 10 LD groups reported by BLINK for Bonneville passage day, tributary arrival day, fork length, and ocean‐age phenotypes, we used sequential MLM models in which cluster‐representative SNPs were included as covariates to estimate *R* ^2^, with SNP covariate addition specified by the order of significance (by *p*‐value) from the BLINK model. As GAPIT does not allow for missing data in covariates, missing genotypes in these sequential MLM models were specified as heterozygotes. In all the GAPIT models, sex was specified as a covariate along with three PCA axes, and in the run timing analyses of BONAFF samples, ocean‐age and fork length were included as covariates as well, with missing data filled in as 1.5 years (*N* = 34) or the median length (*N* = 3), respectively. However, age and length data were not consistently available for the Hood River samples, and so were not included. Sex was assigned based on the genotype of the sex markers. GAPIT MLM models were repeated for the inland and inland + intermediate lineage fish separately, but we did not test intermediate (*N* = 16) and coastal (*N* = 27) lineage fish alone because of insufficient sample size. Following global association analyses, we used nonparametric one‐way analyses of variance (Kruskal & Wallis, 1952), in R to test the association of genotypes for the top three markers, as well as the one (marker nine) previously surveyed by Micheletti, Hess, et al. (2018), with raw migration timing phenotypes in each sub‐basin with sufficient sample size (*N* > 15).

Genotypes are not independently distributed among samples due to linkage, which is a manifestation of the frequency of the haplotypes in which they are found. Another way to examine this is through haplotype association testing of markers in the candidate regions, which we performed in two ways for the complete BONAFF and Hood River datasets and four raw run timing and age‐at‐maturity phenotypes. First, haplotype score statistics were estimated with the package Haplo.stats 1.7.9 (Sinnwell and Schaid, 2005) in R. Haplotype scores are based on the association of the maximum likelihood probabilities of ambiguous haplotypes with the phenotype residuals from a GLM model of nongenetic covariates (here, the same covariates as in GAPIT, with the exception of the kinship matrix). Significance is estimated through permutation of phenotype states; for these analyses, a minimum of 1,000 permutations were run, with permutations continued until the standard error fell below 0.05*p‐value, and only haplotypes with estimated frequency ≥5 observations were scored. Haplotype estimation in Haplo.stats is made using an EM algorithm with progressive marker insertion (here, *N* = 6 SNPs) and a posterior probability trimming threshold for ambiguous haplotype pairs; here, the number of attempts at haplotype estimation was set at 100, with other parameters default (posterior < 1 × 10^−9^, convergence < 1 × 10^−5^). Haplotype scores were calculated on the complete sequence (*haplo.score*), as well as sliding subsets of three markers (*haplo.score.slide*). Haplotype scores were calculated assuming both additive and dominant effects, and given haplotype frequencies inferred by this algorithm relative to sample sizes, we estimated the power to detect significant scores over a range of correlation values. Second, we tested each haplotype individually as a binary marker (pseudo‐SNP) in GAPIT. Haplotype genotypes were inferred using Shapeit v2.r904 (Delaneau, Marchini, & Zagury, 2012). To make haplotype association comparisons more direct, haplotypes were inferred from a pooled BONAFF and Hood River chromosome 28 dataset, although each was tested separately in GAPIT. Unlike Haploview or Haplostats, Shapeit uses a Hidden Markov model (HMM) based algorithm which represents haplotype‐space in a graph to manage complexity, similar to Beagle (Browning & Browning, 2007), but is reported to be faster and more accurate (Delaneau et al., 2012). Shapeit was run with 200 burn‐in and pruning iterations, 5 pruning stages, 500 main iterations, and global recombination rate (rho) of 1 × 10^−7^ to reflect the strong linkage among markers in these regions (see Results). Each individual was coded as having 0, 1, or 2 copies of each alternative haplotype, where all other haplotypes were coded as reference (0), and these were tested as above using the GAPIT MLM model. The kinship matrix and PCA axes for GAPIT analysis of haplotypes were those inferred from the “neutral” SNP dataset.

## 3 RESULTS

### 3.1 Migration timing phenotypes

**Figure 2:**
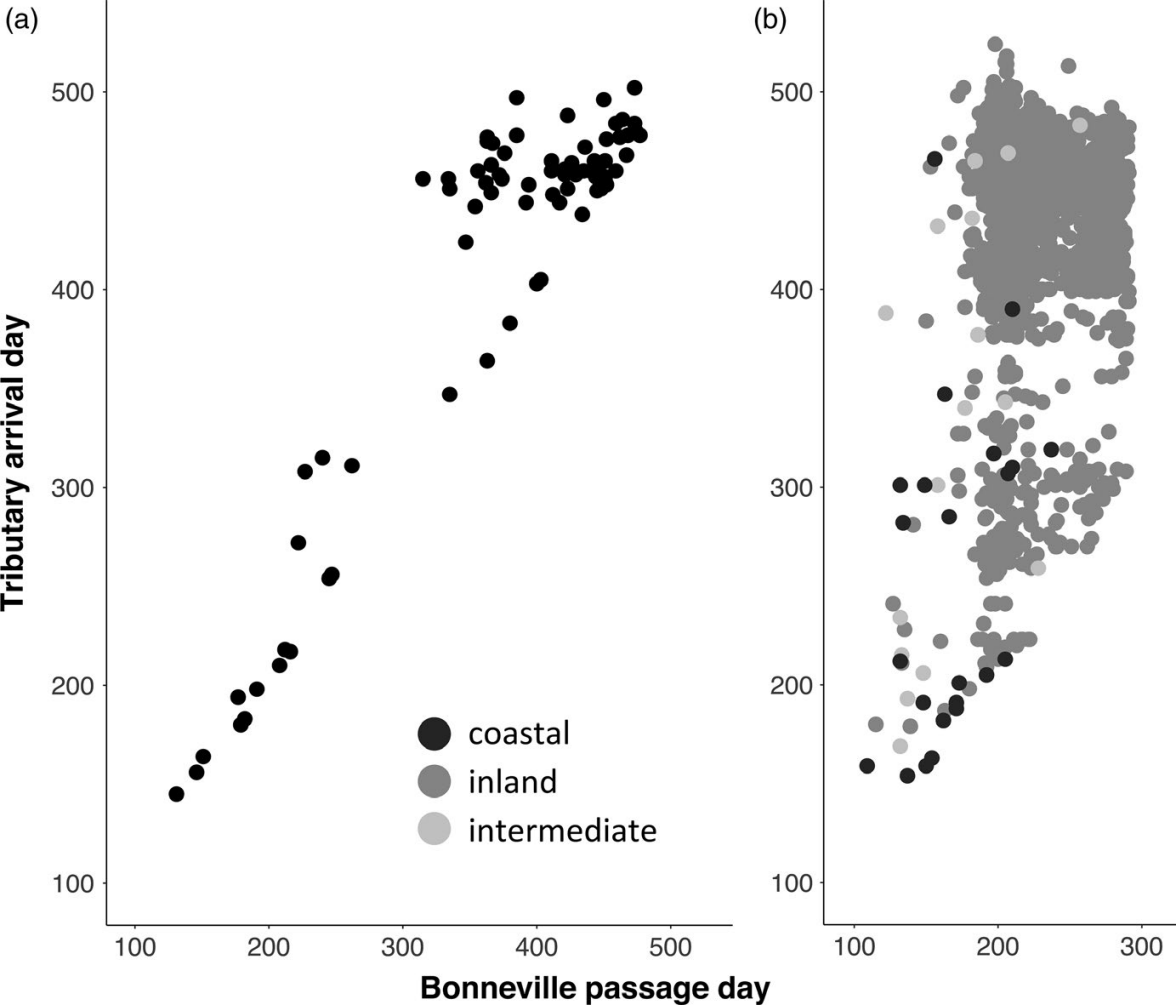
Bonneville passage day and Tributary arrival day (ordinal dates) for Columbia River steelhead (*Oncorhynchus mykiss*). (a) *N* = 77 Hood River steelhead (b) *N* = 1,538 Bonneville Dam Adult Fish Facility (BONAFF) steelhead. BONAFF steelhead are classified by lineage, based on the sub‐basin to which they arrived

For steelhead traveling to the Hood River for which both Bonneville passage and tributary arrival day were known, there was a clear correlation between these days (*R* ^2^ = 0.93, *p* < 1 × 10^−15^; Figure 2). The Hood River dataset also includes a substantial portion of “winter‐run” fish which passed Bonneville Dam at times when sampling does not occur. For the BONAFF samples, as observed by Keefer, Wertheimer, Evans, Boggs, and Peery (2008), there was a strong propensity for inland steelhead passing Bonneville in the fall to overwinter in larger‐order rivers rather than their spawning tributary, but this appeared to also be influenced by destination (Figure 2; Figure S2).

While similar to the trends for the Hood River samples, the overall pattern for BONAFF fish was less distinct (Figure 2; Figure S2). Fish that passed Bonneville Dam before ordinal day 175 (June 24) were mostly destined for lower and middle Columbia tributaries and generally proceeded immediately to the spawning tributary before the end of December (80%; R^2^ for Bonneville passage day and tributary arrival day, 0.49; *p* < 1 × 10^−15^). Steelhead that passed Bonneville Dam after ordinal day 175 were mostly migrating to upper Columbia or Snake River tributaries and arrived in spawning tributaries only after January 1 (87%; *R* ^2^ 0.09, *p* < .001). Interestingly, even during the warmest part of the year during mid‐August and September, ~9% of fish still arrived to the spawning tributary before the end of December, despite challenges imposed by high temperatures and low flows. Conversely, while there was a strong linear trend representing fish that passed Bonneville Dam and arrived to the spawning tributary in the same calendar year, there was a clear disruption in migration trajectory imposed by low winter temperatures and flows (i.e., overwintering) for most fish passing after ordinal 175.

### 3.2 Migratory timing association tests

**Figure 3:**
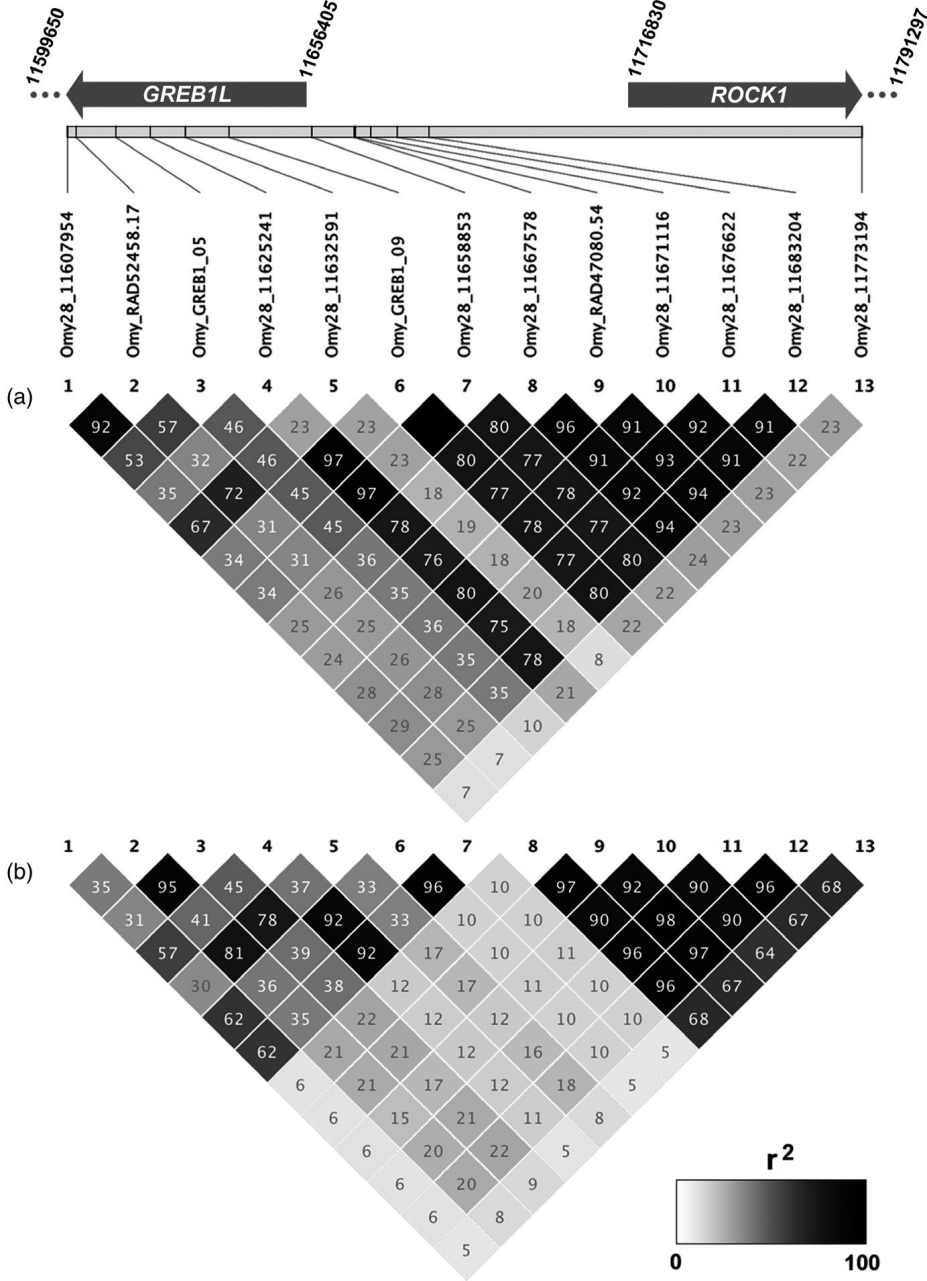
Linkage (*r* ^2^ values) for chromosome 28 candidate markers, and relative spacing of those markers. (a) *N* = 354 Hood River steelhead and (b) *N* = 1,538 Bonneville Dam Adult Fish Facility (BONAFF) steelhead

While both the Hood River and BONAFF samples exhibited two major haplotype blocks in chromosome 28 candidate markers, the borders of these haplotype blocks differed between these two sample sets (Collins et al., 2020). Notably, markers four, six, and seven were linked to the 3′ haplotype block (relative to the top strand) in the Hood River samples, but with the 5′ haplotype block in the BONAFF data (Figure 3), reflecting the underlying difference in haplotype frequencies in each dataset (Table 1, Table S2). Indeed, the Hood River was dominated by haplotypes with different SNP alleles across all 13 markers (Haplotypes I and III; Table 1) as well as recombinants for markers on the ends (e.g., Haplotype IV), and showed a less distinct 5′ marker block. The BONAFF dataset, on the other hand, in addition to Haplotype I, exhibited a high frequency of a recombinant between Haplotypes I and III unlinking the 5′ and 3′ regions between markers 7 and 8 (Haplotype II). We note that haplotype frequencies inferred by Haplostats were very similar to those from Haploview, which was not surprising, considering they both use a variant of the EM algorithm. The haplotype frequencies inferred by Shapeit, on the other hand, with its graph‐HMM algorithm, were more divergent due to the inference of rare distinct haplotypes that Haploview/Haplostats did not identify.

**Table 1:** Haplotypes and their frequencies inferred from genotyped markers across all samples

Haplotypes														BONNEVILLE						HOOD		
														Haploview				Haplostats	Shapeit	Haploview	Haplostats	Shapeit
														All	Interior	Intermediate	Coastal	All	All			
I	G	A	G	G	A	G	C	C	G	T	G	T	A	0.702	0.717	0.094	0.130	0.696	0.631	0.612	0.614	0.534
II	G	A	G	G	A	G	C	T	A	C	T	G	T	0.157	0.161	–	–	0.157	0.115	0.007	0.007	0.005
III	A	C	T	A	G	T	A	T	A	C	T	G	T	0.024	0.011	0.562	0.481	0.025	0.020	0.067	0.067	0.057
IV	G	A	G	G	A	G	C	C	G	T	G	T	T	0.014	0.014	–	–	0.014	0.070	0.109	0.107	0.147
V	G	A	G	G	A	G	C	T	A	C	T	G	A	0.018	0.018	–	0.019	0.018	0.069	0.003	0.003	0.006
VI	G	A	G	G	G	G	C	C	G	T	G	T	A	0.011	0.011	–	0.019	0.010	0.015	0.031	0.031	0.084
VII	G	C	T	G	G	G	C	T	A	C	T	G	T	0.017	0.017	–	–	0.017	0.010	–	–	–
VIII	A	C	G	G	G	G	C	C	G	T	G	T	A	0.001	0.001	–	–	0.001	0.003	0.053	0.053	0.051
IX	A	C	T	G	G	G	C	C	G	T	G	T	A	0.002	0.002	–	–	0.003	0.001	0.049	0.049	0.025
X	G	C	T	G	G	G	C	T	A	T	T	G	T	0.010	0.011	–	–	0.010	0.006	–	0.001	–
XI	G	C	T	G	G	G	C	T	A	C	T	G	A	0.008	0.009	–	–	0.009	0.008	–	–	–
XII	G	C	T	A	G	T	A	T	A	C	T	G	A	0.008	0.002	0.187	0.167	0.007	0.007	–	–	–
XIII	G	A	G	G	G	G	C	C	G	T	G	T	T	0.001	0.001	–	–	0.001	0.001	0.019	0.019	0.009
XIV	A	C	T	A	G	T	A	T	A	C	T	G	A	0.003	–	0.094	0.111	0.003	0.009	0.008	0.008	0.021
XV	G	C	T	G	G	G	C	C	G	T	G	T	A	0.002	0.002	0.031	–	0.002	0.018	0.008	0.008	0.008
XVI	G	C	T	G	G	G	C	T	A	T	T	G	A	0.003	0.003	–	–	0.003	–	–	–	–
XVII	G	A	G	G	A	G	C	C	A	C	T	G	A	0.002	0.002	–	–	0.002	0.000	0.002	0.002	–
XVIII	G	C	T	G	A	G	C	T	A	C	T	G	A	0.002	0.002	–	–	0.002	0.002	–	–	–
XIX	G	A	T	G	A	G	C	C	G	T	G	T	A	–	–	–	–	–	0.003	0.005	0.005	0.041
XX	G	C	T	A	G	G	C	T	A	C	T	G	T	0.001	0.001	–	–	0.001	0.004	–	–	–
XXI	A	C	G	G	A	G	C	C	G	T	G	T	A	–	–	–	–	–	0.001	–	–	0.033
XXII	G	A	G	G	A	G	C	T	A	T	T	G	T	–	–	–	–	–	0.006	–	–	0.002
XXIII	G	C	T	G	A	G	C	C	G	T	G	T	A	–	–	–	–	–	0.003	–	–	0.002
XXIV	G	A	G	G	A	G	C	T	A	T	T	G	A	–	–	–	–	–	0.003	–	–	0.002
I		C	A	G	C	G	G	A	A	A	C			0.637	0.647	0.156	0.314	0.636	0.584			
II		A	C	T	T	A	T	G	G	G	G			0.241	0.239	0.437	0.222	0.241	0.194			
III		A	C	T	T	A	G	A	A	A	C			0.063	0.063	0.406	0.204	0.063	0.113			
IV		C	A	G	C	G	G	G	G	G	G			0.023	0.024	–	–	0.023	0.024			
V		C	A	G	C	G	T	G	G	G	G			0.012	0.012	–	0.222	0.012	0.058			
VI		A	C	T	T	A	T	G	A	G	C			0.011	0.008	–	–	0.011	0.011			
VII		A	C	T	T	A	T	A	A	A	C			0.005	0.005	–	0.019	0.005	0.001			
VIII		C	A	T	T	A	G	A	A	A	C			0.003	0.003	–	–	0.003	0.003			
IX		A	C	G	C	G	G	A	A	A	C			0.002	0.002	–	–	0.002	0.002			
X		C	A	G	C	G	T	A	A	A	C			0.002	0.002	–	–	0.002	0.006			
XI		A	C	T	T	A	G	G	G	G	G			0.001	0.001	–	–	0.001	0.001			
XII		C	A	G	C	G	G	A	A	G	C			–	0.001	–	–	0.001	0.001			
XIII		C	A	G	C	G	G	G	A	G	C			–	–	–	0.019	0.001	<0.001			
XIV		A	A	T	T	A	T	G	G	G	G			–	–	–	–	<0.001	–			
XV		A	C	T	T	A	G	A	G	A	C			–	–	–	–	–	0.001			
XVI		A	A	T	T	A	G	A	A	A	C			–	–	–	–	–	<0.001			
XVII		A	C	T	T	A	T	A	A	G	C			–	–	–	–	–	<0.001			
XVIII		C	A	G	C	G	G	G	A	A	C			–	–	–	–	–	<0.001			

Note

Haplotypes observed in a global minimum of ten individuals for chromosome 28 (above) and all haplotypes inferred from chromosome 25 (below). Shading indicates nucleotide states that differ from the reference haplotypes.

**Figure 4:**
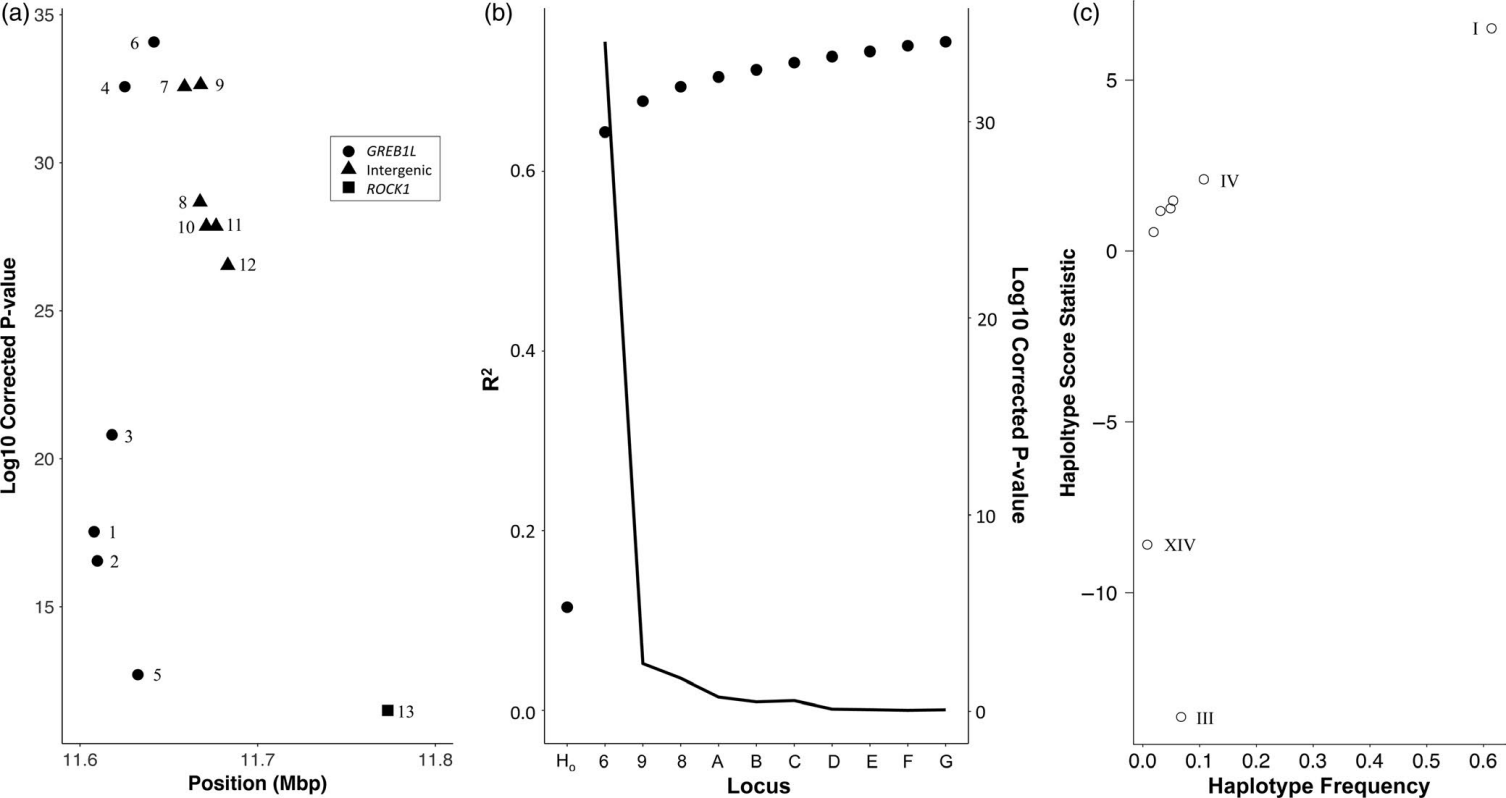
Association of chromosome 28 candidate markers with Arrival day in Hood River steelhead. (a) FDR‐corrected significance values for each SNP marker. Marker numbers correspond to marker details in Table S1 and Figure S3. (b) Phenotypic variance explained (*R* ^2^) and significance of association for most‐significant SNP marker when the previous most‐significant SNP marker is included as a covariate in mixed‐effect models (first two models have no SNP covariates). The line represents the FDR‐corrected significance of association for each marker. Y axis: H_o_: null model; 6, 9, 8: representative markers for significant chromosome 28 linkage groups; A‐G, nonchromosome 28 linkage groups. (c) Haplotype scores for haplotypes with all thirteen markers observed five or more times in Hood River steelhead. A total of eight haplotypes were tested based on minimum frequency in this lineage and the four haplotypes with significant scores (*p* < .05) are labeled following Table 1

For the coastal lineage, Hood River samples, there was a clear trend between genotype at the chromosome 28 candidate markers and both Bonneville passage day and tributary arrival day (Figure S3). Individuals heterozygous for these SNP markers generally exhibited intermediate to late ordinal dates, depending on the marker. The MLM model in GAPIT identified significant associations between these candidate markers and both Bonneville passage day and tributary arrival day, with the latter having more significant association (FDR‐corrected *p* < 1×10^−7^ vs. *p* < 1 × 10^−34^, respectively) but only explaining a slightly higher proportion of phenotypic variation (maximum net *R* ^2^ for model with and without SNP, 0.51 vs. 0.53) than the former (Figure S4, File S6). While all thirteen markers were significant, the most significant markers for tributary arrival day were four, six, seven, and nine (Figure 4). The two most significant SNPs for Bonneville passage day were markers six and nine (File S6). BLINK identified a linkage group with marker six as explaining the most variance in Bonneville passage day and tributary arrival day, and this linkage group was significant for tributary arrival (*p* < 1 × 10^−15^) but not for Bonneville passage (*p* > .07; File S6). For both tributary arrival and Bonneville passage day, the linkage group with marker six explained ~50% of the variance in the data as estimated by the MLM model, while the next most significant linkage group only explained 1.9% to 3.2% of the residual variance when using marker six as a covariate (Figure 4). Nonparametric tests of differences in means of both raw phenotypes by genotype were significant (*p* < 1 × 10^−6^) for all four markers tested (four, six, seven, and nine; Figure [Link], [Link], [Link], [Link]; Table S3).

Haplotype scores estimated from residual phenotype values for Hood River samples in Haplo.stats suggested that the two alternative haplotypes (Haplotypes I and III; Table 1), as well as their recombinants at marker thirteen (Haplotypes IV and XIV; Table 1), were significantly associated with both tributary arrival and Bonneville passage days (Figure 4). We observed no qualitative differences assuming additive or dominant effects of these haplotypes (File S7). Sliding window testing of subhaplotypes showed that significance of haplotype scores peaked at markers six and seven for tributary arrival day, and seven and eight for Bonneville passage day (Figure S9). Testing of Shapeit haplotypes with the GAPIT MLM model demonstrated that the “premature” haplotypes (Haplotypes III and XIV) were significant when contrast to the “mature” haplotype (Haplotype I) (Figure S10) and explained up to 43% and 36% of the variance in tributary arrival and Bonneville passage days, respectively. Power analysis with Haplo.stats showed that for the percent of variation explained by these two haplotypes, power to detect significance was nearly 100% (Figure S9).

**Figure 5:**
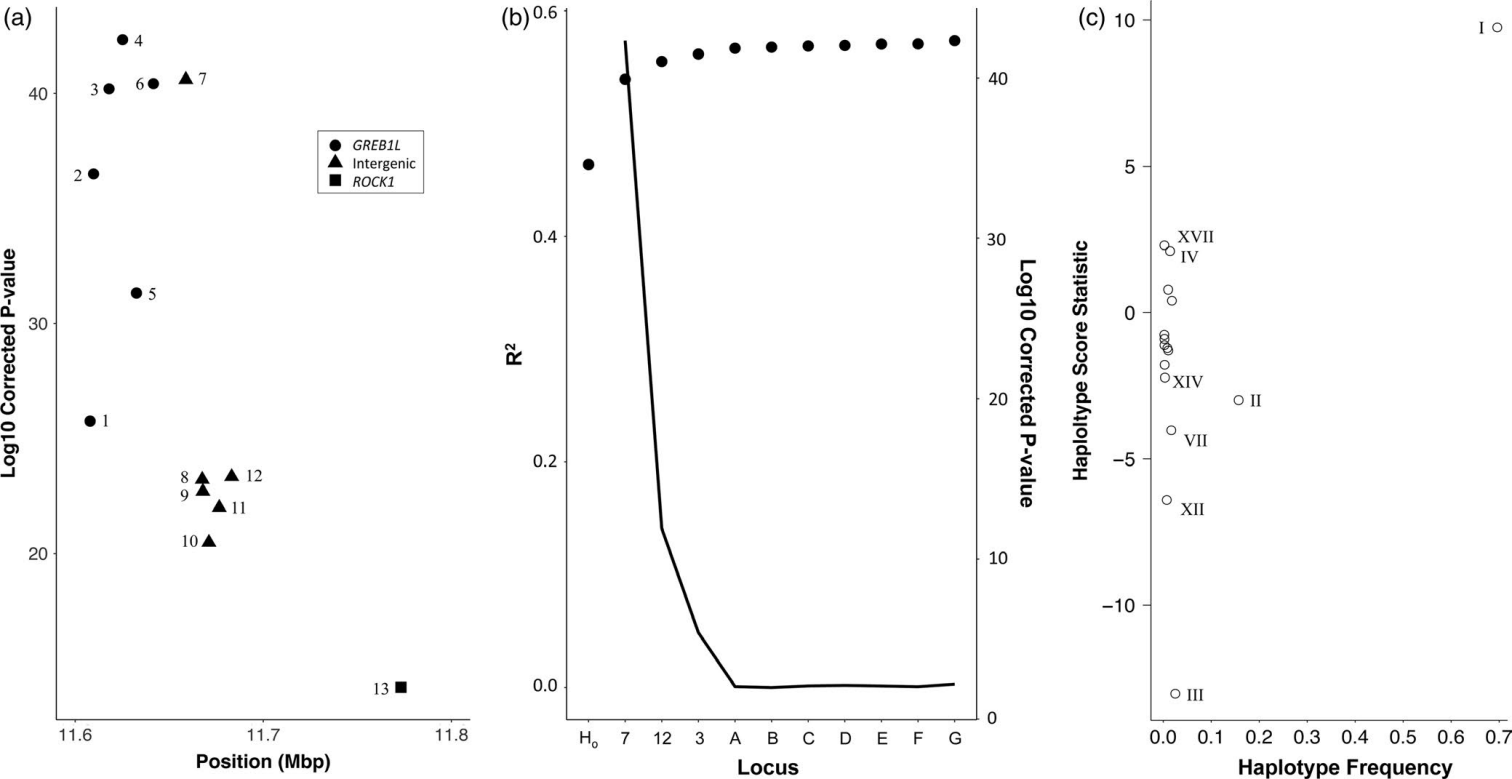
Association of chromosome 28 candidate markers with Bonneville Passage day in Bonneville Adult Fish Facility steelhead. (a) FDR‐corrected significance values for each SNP marker. Marker numbers correspond to marker details in Table S1 and Figure S3. (b) Phenotypic variance explained (*R* ^2^) and significance of association for most‐significant SNP marker when the previous most‐significant SNP marker is included as a covariate in mixed‐effect models (first two models have no SNP covariates). The line represents the FDR‐corrected significance of association for each marker. Y axis: H_o_: null model; 7, 13, 3: representative markers for significant chromosome 28 linkage groups; A‐G, nonchromosome 28 linkage groups. (c) Haplotype scores for haplotypes with all thirteen markers observed five or more times. A total of sixteen haplotypes were tested based on minimum frequency in this lineage and the eight haplotypes with significant scores (*p* < .05) are labeled following Table 1

For BONAFF samples, there was a similar trend in Bonneville passage day according to the chromosome 28 candidate marker genotype, but the trend in tributary arrival day, while still present, was less clear (Figure S11, Figure S12). The individuals homozygous and heterozygous for the “premature” allele, generally exhibited earlier Bonneville passage days than homozygotes with the “mature” allele, across markers, while tributary arrival day for heterozygotes more often overlapped with the latter homozygotes. The GAPIT MLM model reported that the significance of association with Bonneville passage day was higher than for tributary arrival day (min. *p* < 1 × 10^−42^ vs. min. *p* < 1 × 10^−29^), even though the proportion of variance the top marker explained was slightly higher for tributary arrival than Bonneville passage day (*R* ^2^ 0.075 vs. 0.084). Both of these proportions were much smaller than for the Hood River samples (Figures S13, S14, File S8). The most significant SNPs for both raw run timing phenotypes were four, six, seven, and three (Figure 5). We note that, in a GAPIT MLM model using only the 230 “neutral” SNPs, some of these markers were found to be significantly associated with our raw phenotypes, but this was orders of magnitude lower than our candidate markers (Figure S14). When linkage was factored in using the BLINK models in GAPIT, the 5′ linkage group (represented by marker seven) was significant for both raw phenotypes, and this linkage group explained the most variance in the data, despite other candidate and noncandidate linkage groups being significant (Figure 5, File S8).

All of the relative and compound phenotypes of Bonneville passage and tributary arrival day by HUC or sub‐basin, within or across years, had chromosome 28 candidate markers as the most significant SNPs, although with diminished significance and proportions of variance compared to the raw phenotypes (Figure S14, File S8). Nonparametric tests revealed that up to 8 and 5 sub‐basins were individually significant (*p* < .05) for Bonneville passage and tributary arrival day for three top markers (four, six, and seven), respectively, while slightly more sub‐basins were significant for tributary arrival day than Bonneville passage day for marker nine (9 vs. 5, respectively; Figures [Link], [Link], [Link], [Link]; Table S3). Permutations of haplotype scores across phenotypes in the BONAFF data with Haplo.stats indicated that the two most divergent haplotypes (Haplotypes I and III; Table 1) were significantly associated with both Bonneville passage day and tributary arrival day (Figure 5), with GAPIT analysis of the Shapeit haplotypes estimating this contrast explained almost 5% of the variance in the data (Figure S15). Although other recombinant haplotypes were also significant (Figure S15), a power analysis indicated that power was no more than 50% for these haplotypes (R^2^ 0.006–0.014; Figure S16). Sliding window analysis of subhaplotypes indicated that the region of strongest association included marker seven (Figure S16). We observed no qualitative differences assuming additive or dominant effects (File S10).

### 3.3 Age‐at‐maturity phenotypes

There was a strong association between total age estimated from scales (river + ocean duration) and age inferred by parentage assignment (Pearson *R* ^2^ = 0.91, *p* < .001), indicating a relatively clear signal from scale ages (Figure S17). Deviations appear to more frequently overestimate rather than underestimate age based on scales relative to PBT (Figure S17), but it was not possible to tell from these data whether this discrepancy derives more from inaccurate estimates of duration in freshwater or ocean. While some misclassified fish are easily identified based on length (e.g., 1‐ocean fish are not expected to be >70 cm; S. Figure S17), the lower length range of 2‐ocean fish overlaps significantly with 1‐ocean fish, making fish with ocean‐age overestimates difficult to identify by size.

A higher proportion of fish that passed later in the year were 2+‐ocean, for example, 49% vs. 70% before and after August 25, a date previously used to manage Columbia inland steelhead, but 2+‐ocean fish could be seen passing Bonneville throughout most of the sample period, and indeed, a majority of 2+‐ocean fish in this dataset passed between June 30 and August 25 (52%) (Figure S18). Moreover, although a fork length measurement, ≥78 cm, is used by managers to categorize steelhead after June 30, the majority of 2+‐ocean fish were less than this threshold (75%), consistent with previous studies (Copeland et al., 2017; Keefer et al., 2018). Notably, the only fish that stood out from this trend are steelhead returning to the Clearwater River of Idaho (Figure S18), which are known for being disproportionately large at age (Bowersox et al., 2019).

### 3.4 Age‐at‐maturity association tests

**Figure 6:**
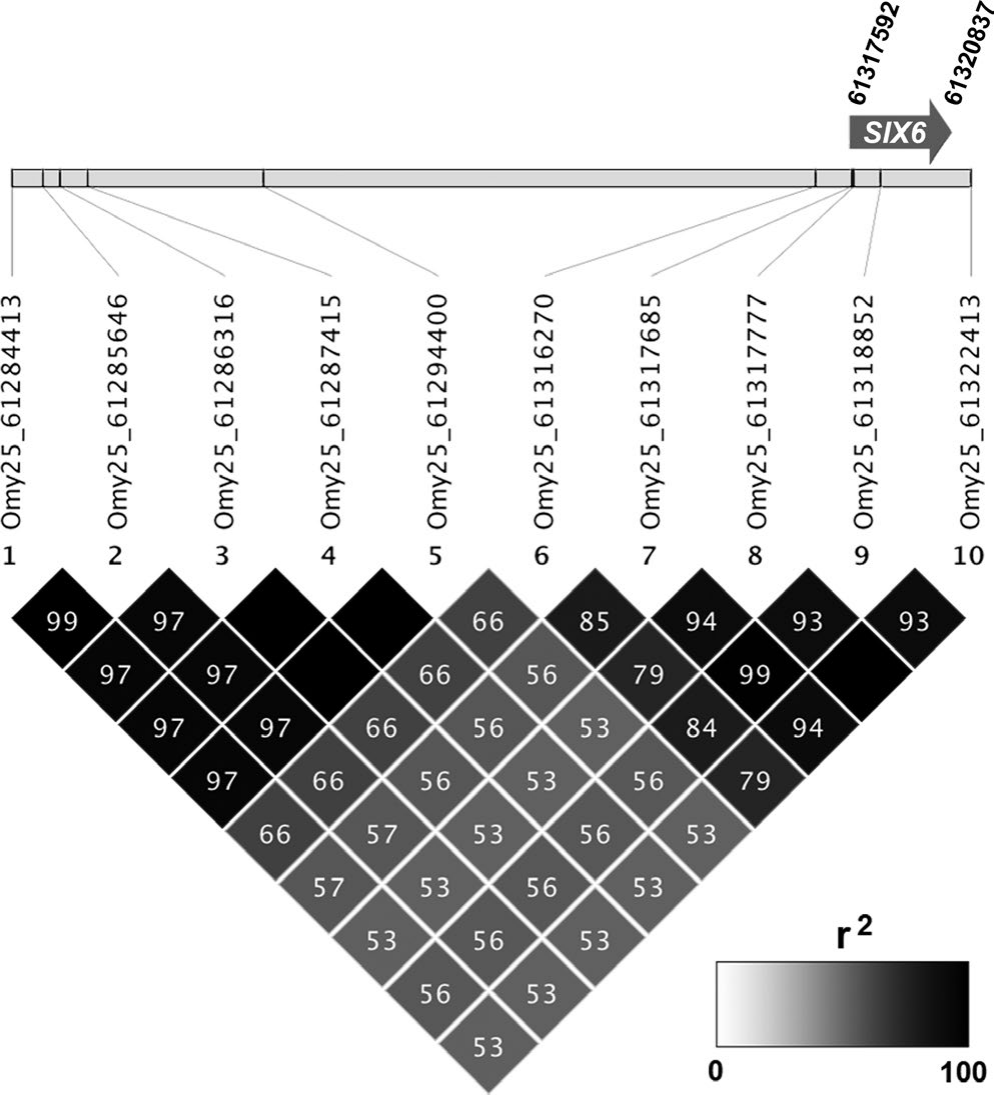
Linkage (*r* ^2^ values) for chromosome 25 candidate markers, and relative spacing of those markers. *N* = 1,538 Bonneville Dam Adult Fish Facility (BONAFF) steelhead

Patterns of linkage in the chromosome 25 candidate markers identified two distinct groups of tightly linked markers, including markers one through five and six through ten (Figure 6). This resulted in a total of 18 distinct haplotypes, ranging in frequency from ~65% in the inland lineage fishes to a single individual, although haplotype frequencies estimated with Shapeit were more divergent than from Haplostats and Haploview (Table 1).

**Figure 7:**
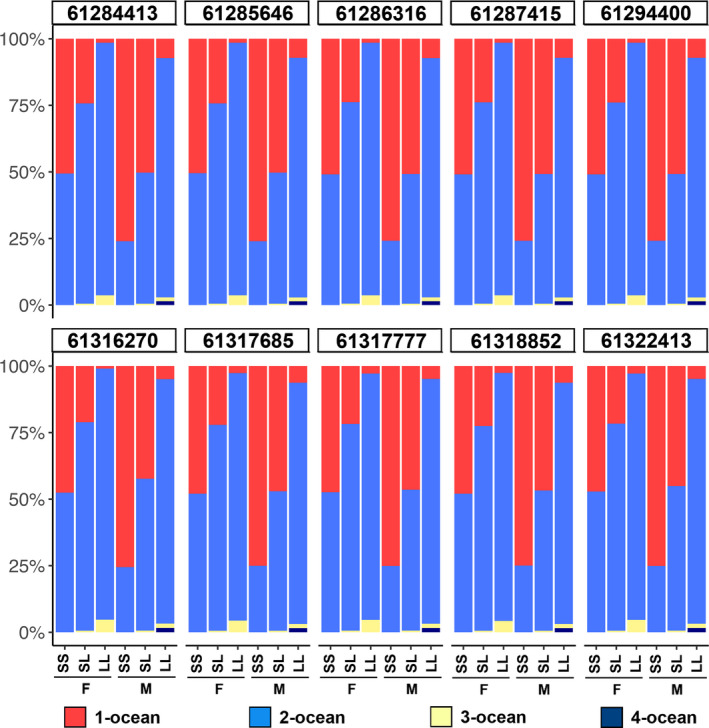
Ocean‐age proportions by sex and genotype for the chromosome 25 candidate markers for Bonneville Adult Fish Facility steelhead. Allele designations: S, shorter length/ocean duration; L, longer length/ocean duration

**Figure 8:**
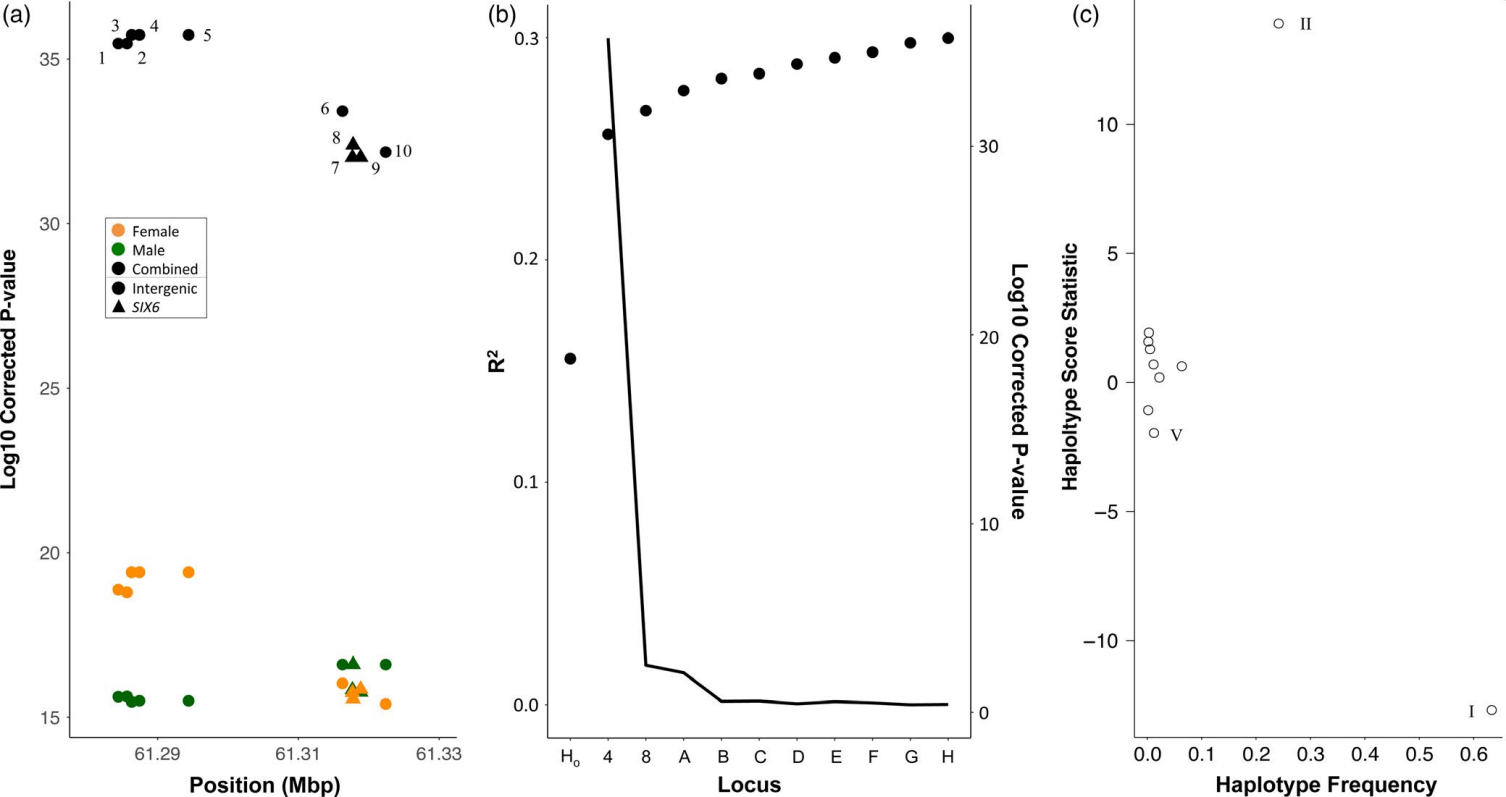
Association of chromosome 25 candidate markers with ocean‐age in Bonneville Adult Fish Facility steelhead. (a) FDR‐corrected significance values for each SNP marker. Marker numbers correspond to marker details in Table S1 and Figure S7. Results are shown for tests on males and females separately and combined. (b) Phenotypic variance explained (*R* ^2^) and significance of association for most‐significant SNP marker when the previous most‐significant SNP marker is included as a covariate in mixed‐effect models (first two models have no SNP covariates). The line represents the FDR‐corrected significance of association for each marker. Y axis: H_o_: null model; 4, 8: representative markers for significant chromosome 28 linkage groups; A–H, nonchromosome 28 linkage groups. (c) Haplotype scores for haplotypes with all ten markers observed five or more times. A total of nine haplotypes were tested based on minimum frequency in this lineage, and the three Haplotypes with significant scores (*p* < .05) are labeled following Table 1

Mean ocean‐age was clearly influenced by genotype of the chromosome 25 candidate markers, as well as by sex, with males showing a stronger effect of chromosome 25 alleles than females of the same genotype. For example, ~50% of males homozygous for the allele associated with shorter ocean durations at marker 1 had one year ocean durations, while only ~25% of homozygous females had one year ocean durations (Figure 7). Based on the association results, hereafter we refer to alleles from candidate markers on chromosome 25 as either “short” (shorter ocean duration and shorter fork length) or “long” (longer ocean duration and longer fork length). Notably, no 3‐ocean or 4‐ocean‐age fish were observed to be homozygous for the “short” alleles in either sex. There was a similar trend for fork length, with homozygous “long” males being 4.9 cm larger than females of the same genotype, on average (Figure S19, Figure S20). The GAPIT MLM model confirmed that the chromosome 25 candidate markers were the most associated with these phenotypes, with the top marker explaining 12.5%, 10.1%, and 3.3% of the variance in fork length, ocean‐age, and total age, respectively (Figure S21, File S11). Not surprisingly, the percentage of variance explained for fork length and ocean‐age was higher in males (17.0% and 13.8%) than in females (11.1% and 9.6%) when tested separately (File S12). In females, as in the combined dataset, markers in the 5′ linkage group were more significantly associated with fork length and ocean‐age, while in males the association was more similar across markers but slightly higher in the 3′ region (Figure 8; Figure S22, File S10). The BLINK model in GAPIT, which considers linkage groups, identified SNPs in the 5′ linkage group, followed by the 3′ linkage group, as being significantly associated with both fork length and ocean‐age, though the majority of variance in each case was explained only by the 5′ linkage group (Figure 8). We observed no substantial difference in degree or pattern of association when considering only inland or inland + intermediate lineage samples (File S13).

Haplotype scores estimated with Haplo.stats, as well as MLM models of Shapeit haplotype genotypes in GAPIT, indicated that the two most divergent haplotypes (Haplotypes I and II; Table 1) were the only ones significantly associated with either fork length or ocean‐age (Figure 8, Figure S23). The GAPIT models estimated that the contrast of these two haplotypes explained 11.3% and 9.1% of the variance in the fork length and ocean‐age, respectively (Figure S24), and power analysis indicated that power for this contrast was approximately 100% (Figure S23). Sliding window analysis of subhaplotypes for both fork length and ocean‐age indicated that the intergenic region just upstream of the *SIX6* gene (markers 5 and 6) showed the most significant association (Figure S23). We observed no qualitative differences in haplotype scores or significance assuming additive or dominant effects of the chromosome 25 haplotypes (File S14).

## 4 DISCUSSION

One critical issue complicating the widespread utilization of genomic markers to predict phenotypes is the confirmation that the same gene(s) underly phenotypes across stocks, and that molecular markers remain similarly predictive (Waples & Lindley, 2018). In the present study, our results validate association of candidate markers with two important phenotypic traits and suggest they may be useful for predicting phenotypic variation for conservation management of steelhead in the Columbia River.

### 4.1 Migration timing

We observed that markers in the chromosome 28 candidate region were significantly associated with aspects of migration timing in both coastal and inland lineage Columbia Basin steelhead, consistent with previous genome‐wide surveys (Hess, Zendt, et al., 2016; Micheletti, Hess, et al., 2018; Prince et al., 2017). In both cases, markers in the same subregion showed the strongest association, indicating that markers in the upstream portion of the *GREB1L* gene (closer to the transcription start site) and intergenic region immediately adjacent were most predictive of adult migration timing. Similarly, haplotypes containing most or all of the “premature” or “mature” alleles (e.g., Haplotypes I, III, IV, and XIV) showed the same pattern of association, although haplotype frequencies provided reduced power to predict phenotypes except for all but the most common haplotypes. However, the degree of association with aspects of adult migration timing differed greatly between coastal lineage, for example, Hood River fish, and inland lineage fish, which constitute the majority of BONAFF samples. While candidate markers from the chromosome 28 region explained roughly 50% of the variance in migration timing of coastal lineage steelhead, with heterozygotes exhibiting an intermediate to late timing for both Bonneville passage and tributary arrival day, these same genotypes explained less than 10% of phenotypic variation for either trait in inland steelhead.

Dominance or co‐dominance of contributing alleles directly influence heterozygotes and fish with these genotypes were evaluated to examine adult migration phenotypes. As in the Hood River samples, heterozygotes in the BONAFF dataset exhibited Bonneville passage days that were often intermediate to either homozygote. Tributary arrival day for heterozygotes, on the other hand, showed greater overlap with homozygotes for the “mature” allele, arriving later in the year than homozygous “premature” fish, a pattern also seen in the Hood River data. While this may superficially suggest that Bonneville passage and tributary arrival day exhibit different expression patterns (additive vs. dominant), neither showed any improved haplotype association patterns with alternative dominance models. It appears likely, then, that although heterozygotes often initiate migration earlier than homozygous “mature” individuals, they migrate too late in the season to overwinter at their spawning destination, thus arriving in a similar time period as homozygous “mature” migrants. It is curious nonetheless to note that despite this overall pattern, there are cases of individuals that pass Bonneville Dam late in the fall (typically homozygous “mature” migrants), but still manage to migrate quickly enough to arrive to some of the most distant sites in the basin (e.g., middle Clearwater River) before the onset of the lowest winter temperatures. Conversely, one of the major concerns regarding heterozygotes is their exposure to high water temperatures during summer migration and potential for reduced fitness (Quinn et al., 2016). Whether or not these intermediate‐to‐late migrating heterozygous individuals can serve as a buffer for poor reproductive success in premature individuals, that is as a reservoir for “premature” alleles (Prince et al., 2017), remains unclear.

Large discrepancies in the variance in run timing explained by chromosome 28 variation between lineages may be related to long migration distance and environmental effects that result in phenotypic plasticity for inland steelhead (Keefer, Boggs, et al., 2008; Micheletti, Hess, et al., 2018). Specifically, the distance and number of hurdles that steelhead migrating to the Columbia River inland have to traverse to reach their spawning destination increases the opportunity for environmental effects to alter migration phenology imbued by chromosome 28 variation. It is evident that inland steelhead initiate migration to freshwater in a gonadal state that would be considered premature (Busby et al., 1996; Hess, Zendt, et al., 2016; Quinn et al., 2016), but they also exhibit variation for timing of arrival for spawning (Keefer, Boggs, et al., 2008; Micheletti, Hess, et al., 2018). While early freshwater entry timing would predict extremely high frequency of premature alleles in the inland lineage if that were the associated phenotype, the actual genetic variation at chromosome 28 has a much higher frequency of mature alleles for inland steelhead as shown in this study and previous work (Collins et al., 2020; Micheletti, Hess, et al., 2018). In fact, genetic variation at candidate markers is significantly associated with phenotypic variation in timing of tributary arrival for spawning (Micheletti, Hess, et al., 2018). This is consistent with the putative gene functions for *GREB1L* as a retinoic acid receptor coactivator and *ROCK1* as a RhoA‐dependent kinase strongly expressed in reproductive and renal tissues (Brophy et al., 2017; De Tomasi et al., 2017; Mizuno et al., 2013; Nakagawa et al., 1996; Oviedo et al., 2011; Sanna‐Cherchi et al., 2017). It remains an intriguing possibility that different variants within this chromosome 28 region have separate effects on these different aspects of adult migration timing, freshwater entry, and tributary arrival timing (Ford et al., 2020; Thompson et al., 2019). Potentially consistent with this, we observed that while the markers in the 5′ linkage group (four, six, and seven) were more often significant in nonparametric tests for Bonneville passage day than tributary arrival day, the opposite was true for marker 9, in the 3′ linkage block. However, it is notable that timing of freshwater entry coordinates with reproductive timing in that our data show a significant correlation between passage at Bonneville Dam and tributary arrival dates. This indicates that steelhead initiate migration to match their reproductive timing at spawning grounds in both lineages, but inland fish are forced to deal with greater environmental variation that results in greater phenotypic variation than in the coastal lineage.

We note at least two caveats in phenotypic data inferred from PIT tags. First, steelhead may hold their freshwater migration within the initial 234 km of the Columbia River prior to being detected at Bonneville Dam, reducing the accuracy of this proxy for freshwater entry timing. Second, PIT arrays do not detect every passage event, making it difficult to ensure that individuals reached their spawning tributary destination rather than exhibiting migratory behaviors such as taking temporary refuge, “overshooting,” or attempting iteroparity (Keefer & Caudill, 2014; Keefer, Boggs, et al., 2008; Keefer et al., 2018, 2008), introducing inaccuracy to tributary arrival timing phenotypes. However, setting aside the differences in collection methods may have on the accuracy of tributary arrival day for the present samples, these caveats should apply similarly to coastal and inland steelhead, suggesting the differences noted here are likely to be real.

Despite the similarity in the genetic basis for migration timing in Chinook salmon (*Oncorhynchus tshawytscha*) and steelhead (Prince et al., 2017), there appear to be subtle differences in the patterns of association between these species. Janowitz‐Koch and Narum (2020) recently examined variation in the *GREB1L‐ROCK1* region of chromosome 28 relative to arrival time in Chinook salmon from three Columbia River lineages: coastal, interior ocean type, and interior stream type, which all exhibit clear differences in arrival timing for spawning (Narum, Genova, Micheletti, & Maass, 2018). That study discovered slightly stronger associations with markers in the 5’ region of *ROCK1* and intergenic regions just upstream of that gene than with *GREB1L*. Interestingly, these authors also noted that association was stronger in coastal and interior ocean‐type Chinook salmon (max net *R* ^2^ 29% and 78%, respectively) than for interior stream‐type (5%), from which they inferred that stream‐type Chinook, which migrate in spring and summer, are more susceptible than their counterparts to interannual environmental variation that moderates migration phenology. In a similar study, Thompson et al. (2019) found that the two SNPs most strongly associated with run timing were in the intergenic region between *GREB1L* and *ROCK1*. Along with the present results highlighting the upstream portion of *GREB1L* and the adjacent intergenic region, this may suggest that the causative variants lie in a regulatory region, which affects gene expression at one or both genes, although which of these genes may be more important in either steelhead or Chinook salmon remains uncertain. Notably, Thompson et al. (2019)also speculated that their chromosome 28 markers were more predictive of freshwater entry time than tributary arrival time, since some individuals, particularly heterozygotes, tended to hold in freshwater downstream of spawning tributaries, delaying final arrival and diminishing the strength of association with this aspect of run timing. Nonetheless, it remains to be seen what the strength of association with migration timing is for these markers across the range of both species, and of which migration aspects they are most predictive.

### 4.2 Age‐at‐maturity

Consistent with recent genomic surveys, we found that variation in markers on chromosome 25 in or near the *SIX6* gene was significantly associated with both ocean‐age and fork length, and to a lower degree, total age. The latter is not particularly surprising, given that variation in years that juveniles spend in freshwater tend to be more variable and difficult to estimate in scale patterns than years spent in the ocean (McNicol & MacLellan, 2010). Further, ocean‐age and fork length are strongly correlated since the greatest amount of growth occurs for steelhead while feeding in highly productive marine environments (Brannon et al., 2004; Busby et al., 1996). Similarly, because the margin of error on ocean‐age (±1 year) is much larger relative to overall variance than the precision for fork length, it should not be surprising that associations with fork length were generally stronger and more significant than ocean‐age, irrespective of which trait is more directly under the influence of the causative variant(s). However, the much weaker results of total age suggest little to no association of these candidate markers with freshwater age. Intriguingly, the markers most associated with both ocean‐age and fork length were located in the intergenic region upstream of the *SIX6* gene rather than those in the gene itself. Indeed, the sliding window analysis of subhaplotypes suggested that the intergenic region had the strongest association for both fork length and ocean‐age. This suggests that the causative variation with which these markers are linked lies in a regulatory region that effects expression of the *SIX6* gene. This association in steelhead shares some similarities with the genetic basis for this same trait in Atlantic salmon where SIX6 has been identified as a candidate gene (Barson et al., 2015; Sinclair‐Waters et al., 2020). Studies of *SIX6* in Atlantic salmon demonstrating tissue and early‐development stage‐specific expression differences for this genes are important revelations (Kurko et al., 2019), but even with these insights, confirming the manner in which this candidate gene mediates age‐at‐maturity will be a significant challenge. Although it is now clear that variation in this genomic region has large effects on adult phenotypes, albeit effects that may be stock‐specific in pattern and degree, the traditional inference that these traits are subject to environmental and developmental thresholds is likely still true (Copeland et al., 2017; Kendall et al., 2015; Thorpe, Mangel, Metcalfe, & Huntingford, 1998), and it is unclear in which developmental stages or environments these thresholds operate.

We also identified a sex‐dependent pattern of association with ocean‐age in male versus female steelhead. Although we are not the first to observe this sex‐dependent pattern in salmonids, Barson et al. (2015) interpreted the patterns of sex‐biased association with genotypes of Atlantic salmon as “sex‐dependent dominance,” which implies the largest changes in expression are observed in heterozygotes (see also Aykanat et al., 2019). Here, while heterozygotes did exhibit distinct phenotypic ratios, they directly followed those in homozygotes, implying that there may be one or more additional and potentially sex‐linked genes that mediate the effects of variation in the *SIX6* region (sex‐dependent epistasis). Moreover, sex‐dependent life history traits are not uncommon in salmon, and steelhead in particular. Another well‐known pattern is for the predisposition of males to forego anadromous migration (Brannon et al., 2004), a pattern which may represent a sex‐dependent expression of variation in inverted regions of chromosome 5 (Pearse et al., 2019), and indeed, females outnumbered males in our dataset of anadromous fish by 1.67 times. Overall, the observation of sex‐dependent effects on age‐at‐maturity in addition to an implication of the *SIX6* region in both European Atlantic salmon and Columbia River steelhead is remarkable given this gene has not been reported in a more closely related species to steelhead such as Chinook salmon (McKinney et al., 2020; Micheletti & Narum, 2018; Charles D. Waters et al., 2018).

Sexually antagonistic selection is a common explanation for trade‐offs in gene expression and development of distinct phenotypic states in males and females (Cox & Calsbeek, 2009). Indeed, in salmonids there is ample reason to expect that males and females may experience different selective forces impacting thresholds for life history traits (e.g., Ohms, Sloat, Reeves, Jordan, & Dunham, 2014), such as when to initiate spawning migrations. While male steelhead compete with other males for mating opportunities, an interaction in which size is often advantageous (Foote, 1989; Quinn & Foote, 1994), female steelhead compete indirectly for access to adequate habitat for redd construction (Foote, 1990). Although fecundity is strongly correlated with size in females (Beacham & Murray, 1993), and it would seem that size is generally advantageous in both sexes, the energetics of achieving size are likely strongly distinct between steelhead sexes (Ohms et al., 2014; Quinn, Seamons, Vollestad, & Duffy, 2011). Since females will usually invest much more in egg development than males in testicular development, allowing males to put more effort into somatic growth, the developmental thresholds for when an adequate size has been achieved may also be distinct (Thorpe, 2007). As such, a sex‐linked modifier of the *SIX6* gene's effects on age at return migration would help coordinate male and female development with selective forces of differing degrees or direction. While the sex chromosomes in salmonids are generally not morphologically distinguishable, differing largely in the presence/absence of the male‐associated sdY locus (Yano et al., 2012), the rates of recombination appear to be strongly constrained in males relative to females (Lien et al., 2011; Pearse et al., 2019). If so, this could protect male and female‐specific alleles that modify the underlying effects of *SIX6* on thresholds for age and size at return migration, reducing antagonism in sex‐distinct selection pressures. Consistent with this, McKinney et al. (2020) recently observed Y‐chromosome haplotypes in Chinook salmon that were associated with different mean sizes and ages at return migration across populations. Although, it is not yet clear that there is a common genetic basis for age‐at‐maturity in Pacific salmon, the implication of modifiers for age and size at first reproduction on the sex chromosomes is consistent with the present data.

In addition to individual fitness, diversity in age‐at‐maturity is among the most important life history traits increasing the resilience of salmonid populations (Moore et al., 2014). Multiple age classes returning each spawn year provide a demographic buffer against poor fitness due to high mortality in either juvenile or adult migration in any single year (Keefer et al., 2018; Moore et al., 2014). In steelhead, female iteroparity may serve a similar role by decreasing the demographic effects of recruitment failures from one or a few years (Keefer, Wertheimer, et al., 2008). Moreover, steelhead with distinct age and size at return migration face distinct pressures. While size is expected to be an advantage in traversing the many barriers separating distant spawning tributaries in the Columbia basin, older, summer‐run steelhead overlap in space, time, and size with fall Chinook salmon, for which there is an active gillnet fishery in the lower and middle Columbia River mainstem. Because these 2+‐ocean steelhead, some of which are returning to ESA‐protected Snake River populations, are particularly susceptible to this fishery, the catch limits of steelhead can often restrict the gillnet fishery targeting Chinook salmon (Copeland et al., 2017). However, as the correlation between size, ocean‐age, and population composition appears to have become less clear over time (Bowersox et al., 2019; Copeland et al., 2017; Keefer et al., 2018), we encourage continued investigation of the utility of the chromosome 25 markers examined herein, which appear closely tied to the biological mechanisms for predicting size and age composition, to assist in sustaining life history diversity in these populations.

## 5 Conclusions

Our data show that chromosome 28 variation in the *GREB1L‐ROCK1* region was predictive of multiple aspects of migration timing but in varying degrees for different lineages and stocks of Columbia River steelhead. Additionally, this study shows chromosome 25 variation near the *SIX6* gene was clearly associated with ocean‐age, particularly for males in all stocks and lineages examined in the Columbia River. However, we suggest that stock‐specific examinations may be useful to clarify the degree to which variation in chromosome 28 is predictive of run timing phenology and the linkage patterns that mediate which markers will be most useful for this purpose. As we saw here, genomic background, haplotype frequency, and migration distance may influence apparent associations with molecular markers in an idiosyncratic manner, and more stock‐specific data will be useful to identify the most locally predictive markers and their degree of correlation. Similarly, while there appears to be a deterministic degree of association with ocean‐age for several chromosome 25 markers in Columbia River steelhead, this may not apply to steelhead throughout their full range. For both phenotypes explored in this study, additional scrutiny of polygenic signals is warranted to determine whether other genes affect these traits differently by sex or population.

Our data underscore the assertion that, even where the same genomic region may be expected to strongly influence life history trait variation among conspecifics, the patterns of association are likely to be environmentally mediated and spatially variable, and careful genetic surveys may be necessary before traits can be predicted accurately from genotypic data (Waples & Lindley, 2018). However, we anticipate there may be management applications for these markers in the near future to preserve underlying genetic variation that maintains phenotypic variation necessary for long‐term persistence of this species (Schindler et al., 2015), including, for example, the monitoring of candidate gene frequencies in returning stocks to inform conservation measures needed to maintain an adequate balance of underlying genetic variation for these traits.

## Supporting information

Fig S1

Fig S2

Fig S3

Fig S4

Fig S5

Fig S6

Fig S7

Fig S8

Fig S9

Fig S10

Fig S11

Fig S12

Fig S13

Fig S14

Fig S15

Fig S16

Fig S17

Fig S18

Fig S19

Fig S20

Fig S21

Fig S22

Fig S23

Fig S24

Table S1

Table S2

Table S3

File S1

File S2

File S3

File S4

File S5

File S6

File S7

File S8

File S9

File S10

File S11

File S12

File S13

File S14

## Data Availability

Data for this study are available at https://doi.org/10.5061/dryad.mpg4f4qwt. Computer code is available in the supplemental files or at https://github.com/stuartwillis/Progeny_convert_haplotype_genotype.
