# Supplementary figures and images for: Steelhead (Oncorhynchus mykiss) lineages and sexes show variable patterns of association of adult migration timing and age‐at‐maturity traits with two genomic regions
Source: Evol Appl. 2020 Aug 27;13(10):2836–56. doi: 10.1111/eva.13088 (PMC7691471; doi:10.1111/eva.13088)

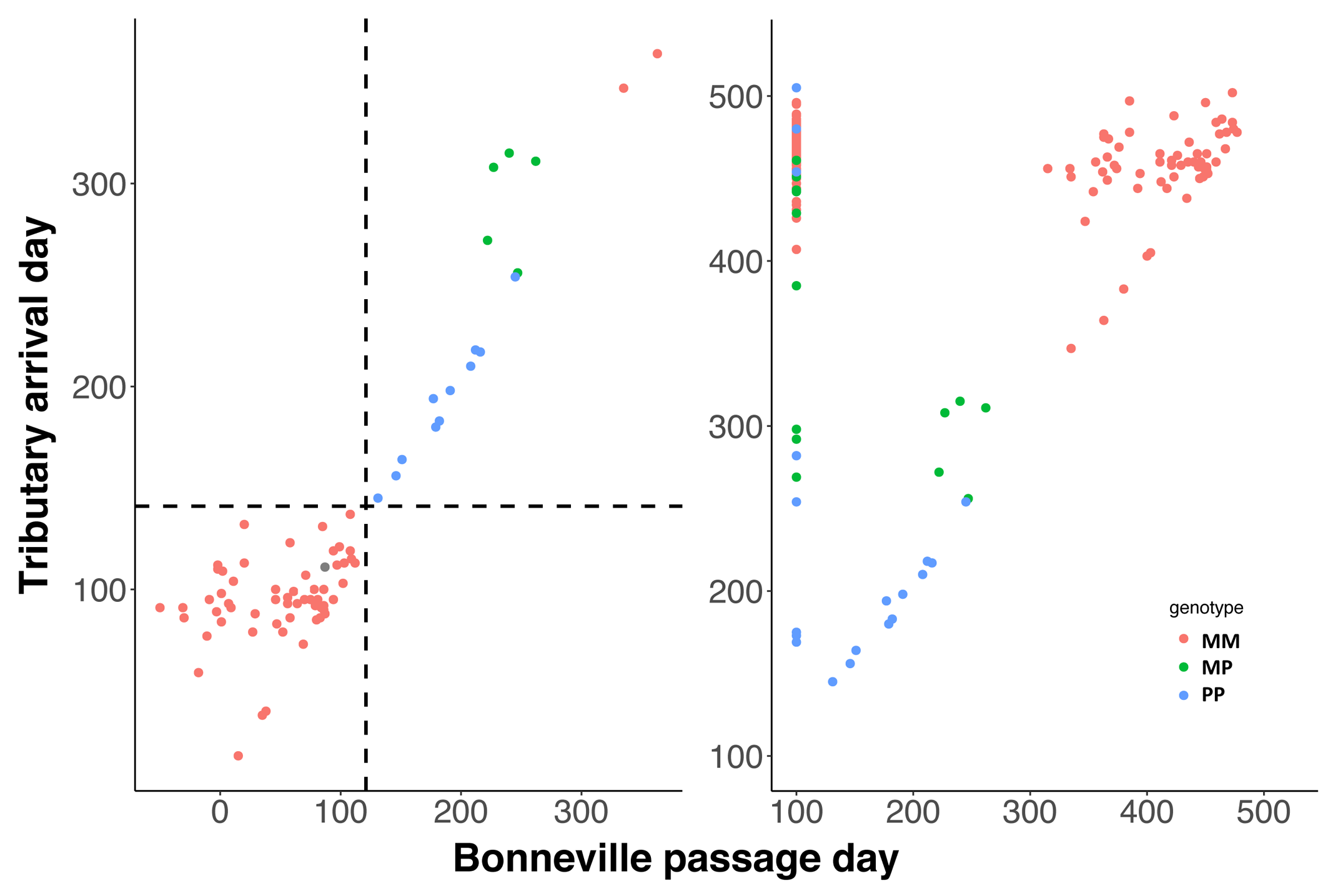

Supplement: Supplementary file 1 — Fig S1 [file EVA-13-2836-s001.tif]

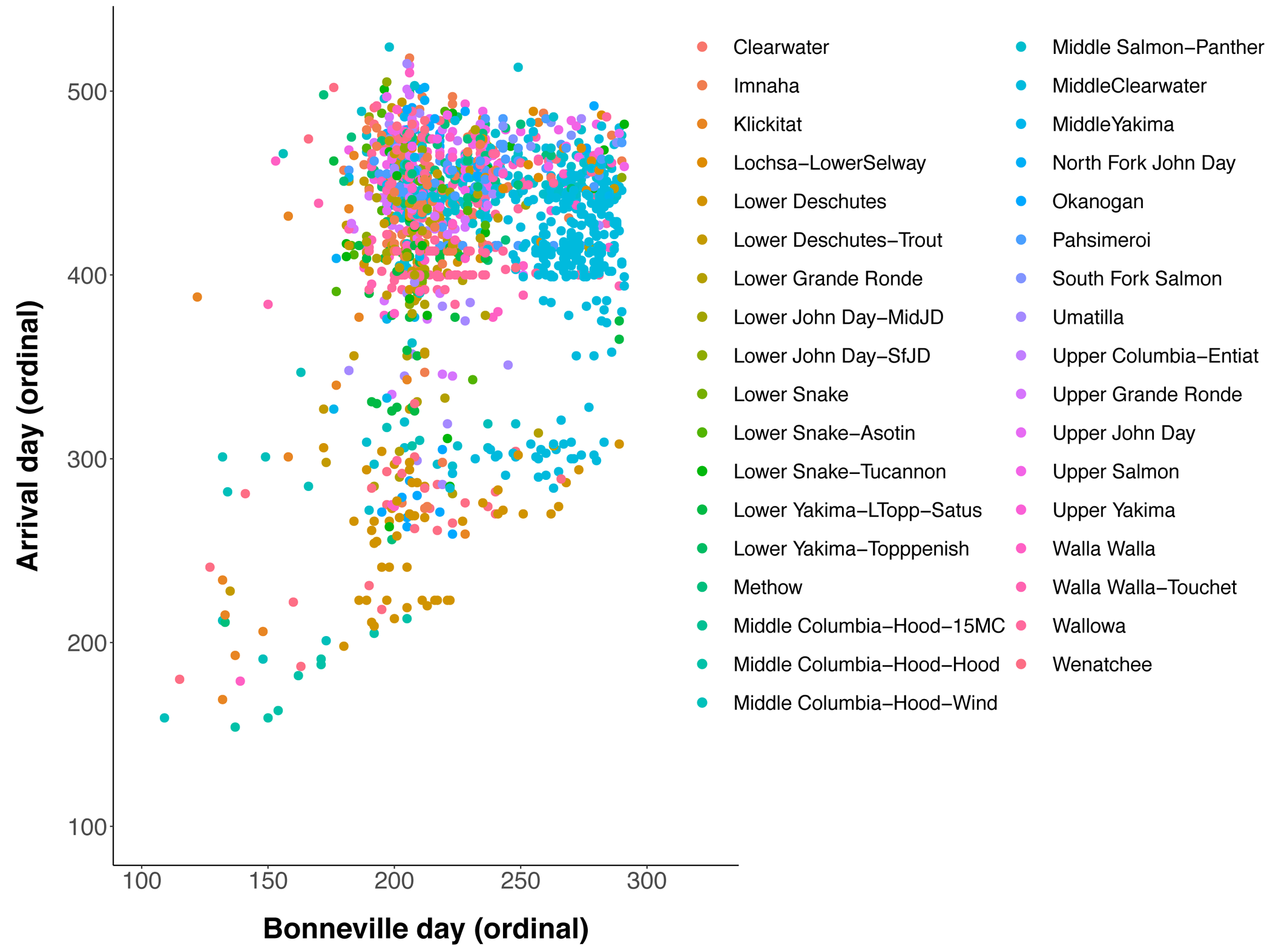

Supplement: Supplementary file 2 — Fig S2 [file EVA-13-2836-s002.tif]

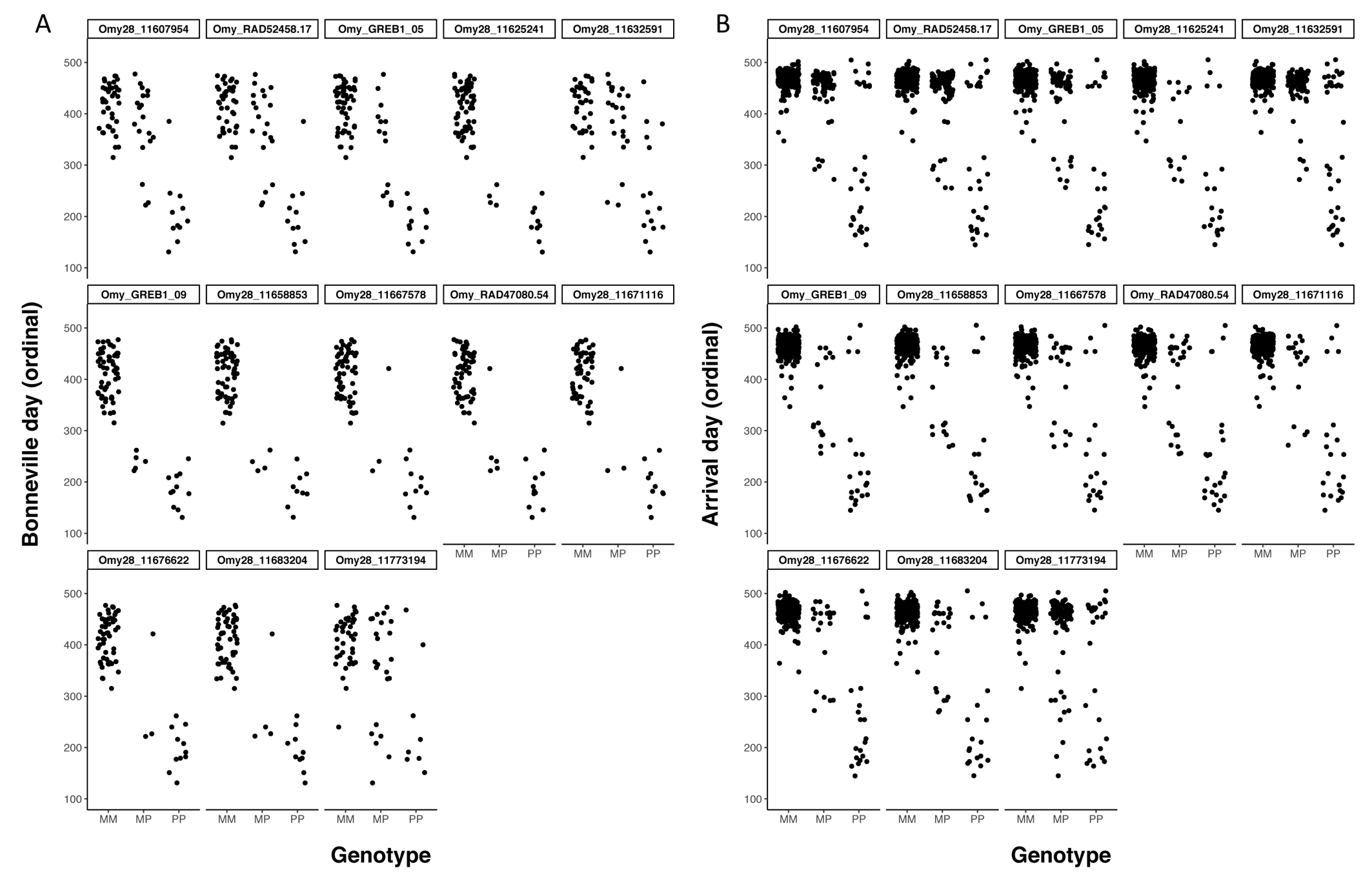

Supplement: Supplementary file 3 — Fig S3 [file EVA-13-2836-s003.tif]

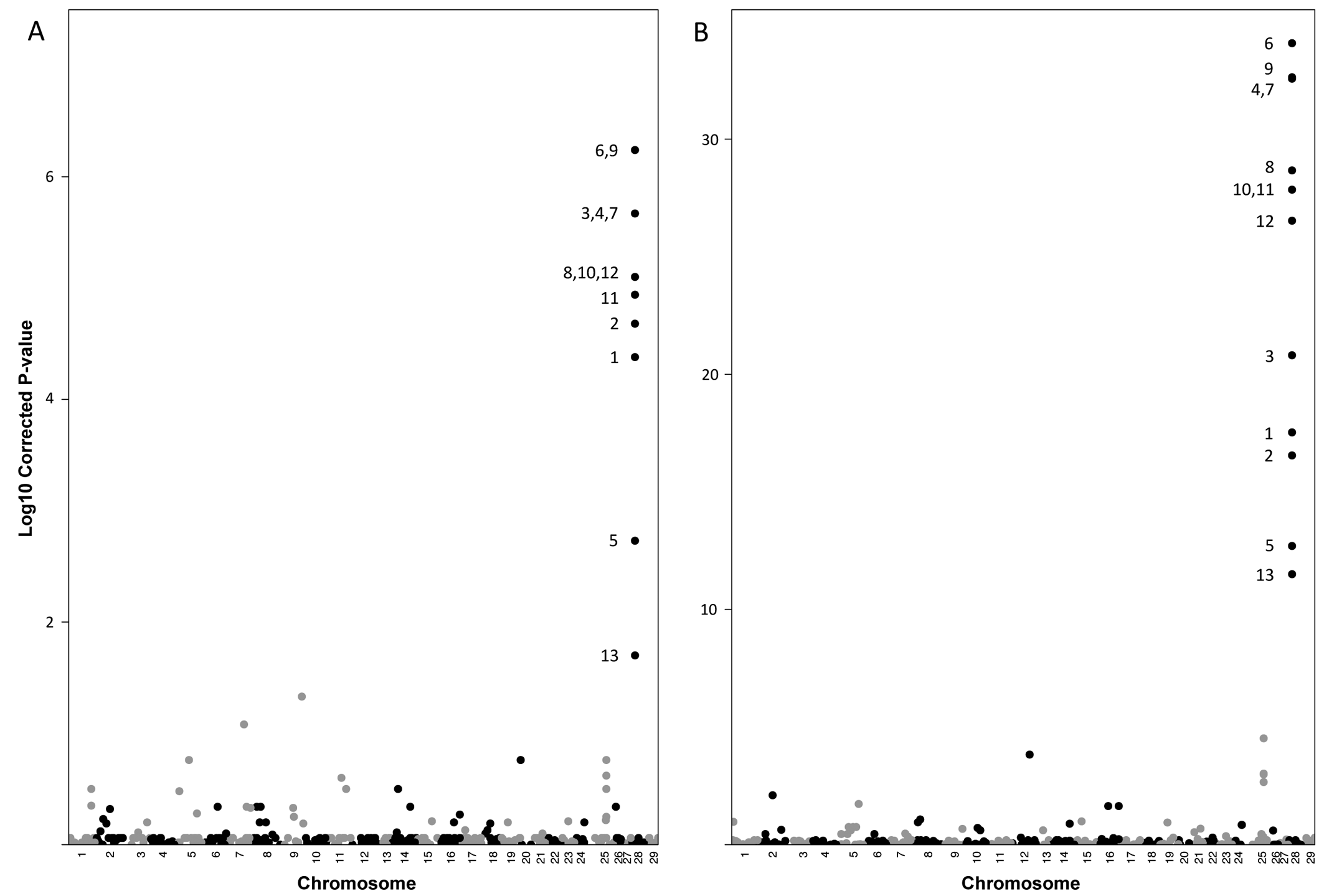

Supplement: Supplementary file 4 — Fig S4 [file EVA-13-2836-s004.tif]

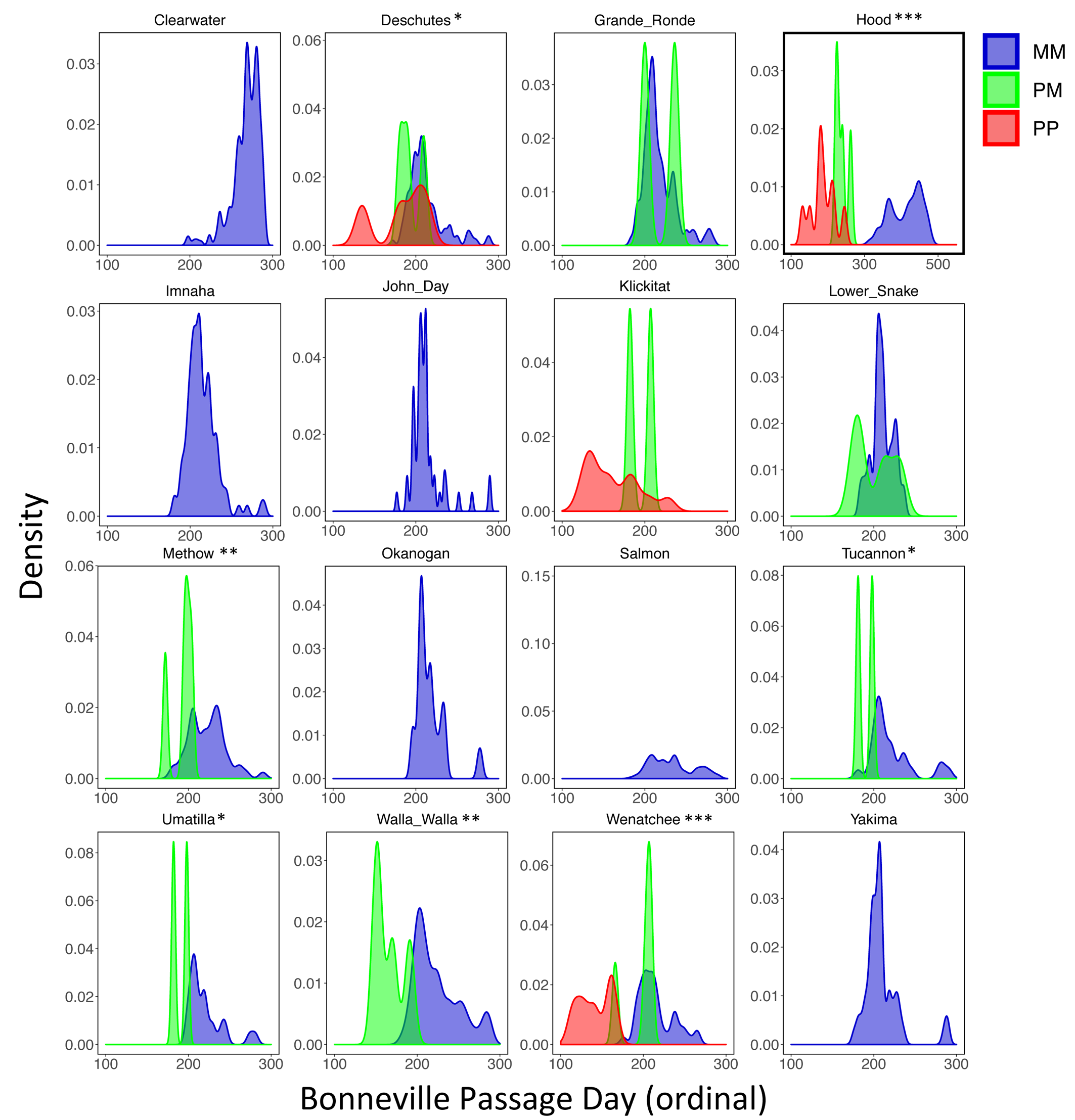

Supplement: Supplementary file 5 — Fig S5 [file EVA-13-2836-s005.tif]

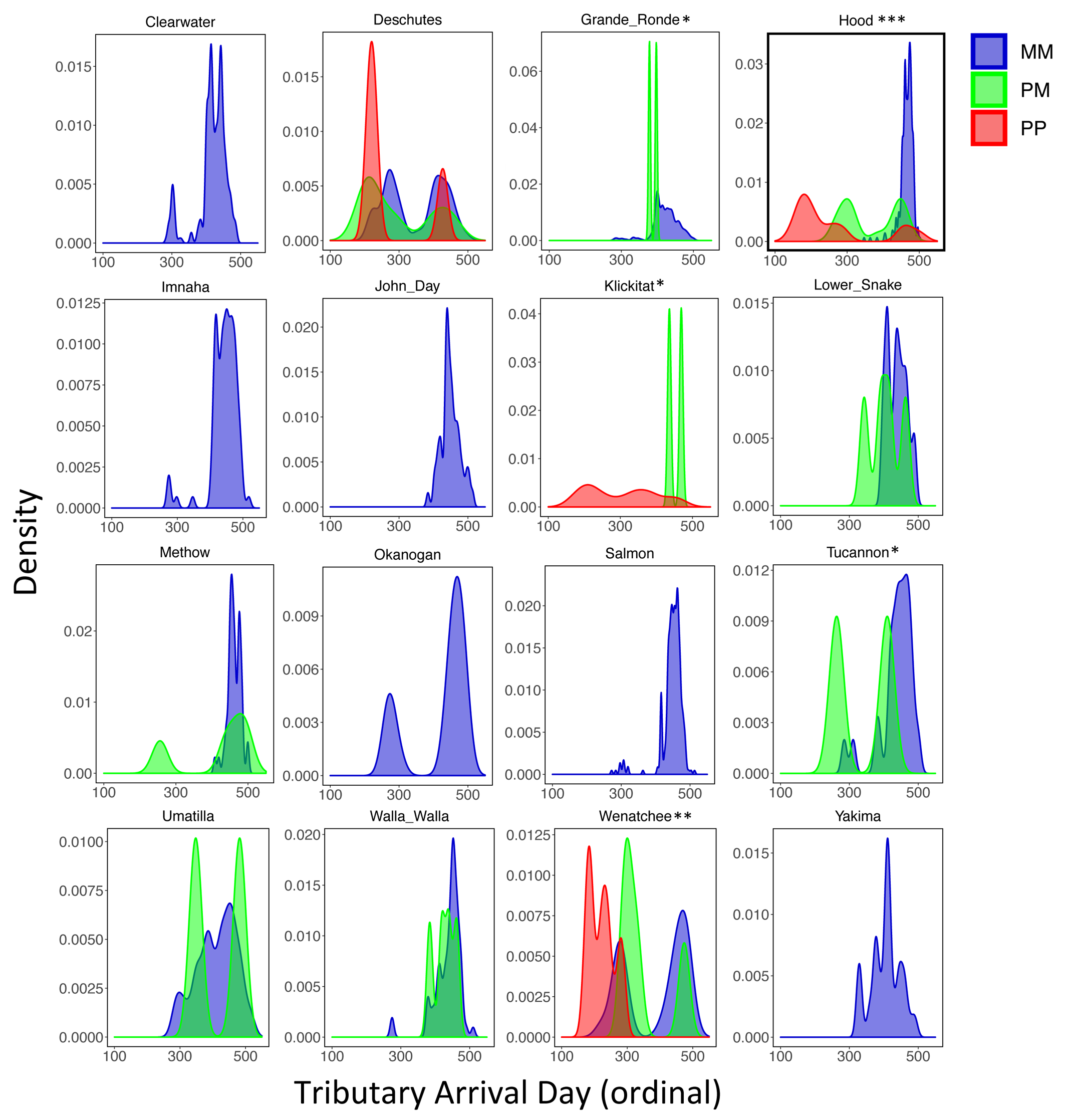

Supplement: Supplementary file 6 — Fig S6 [file EVA-13-2836-s006.tif]

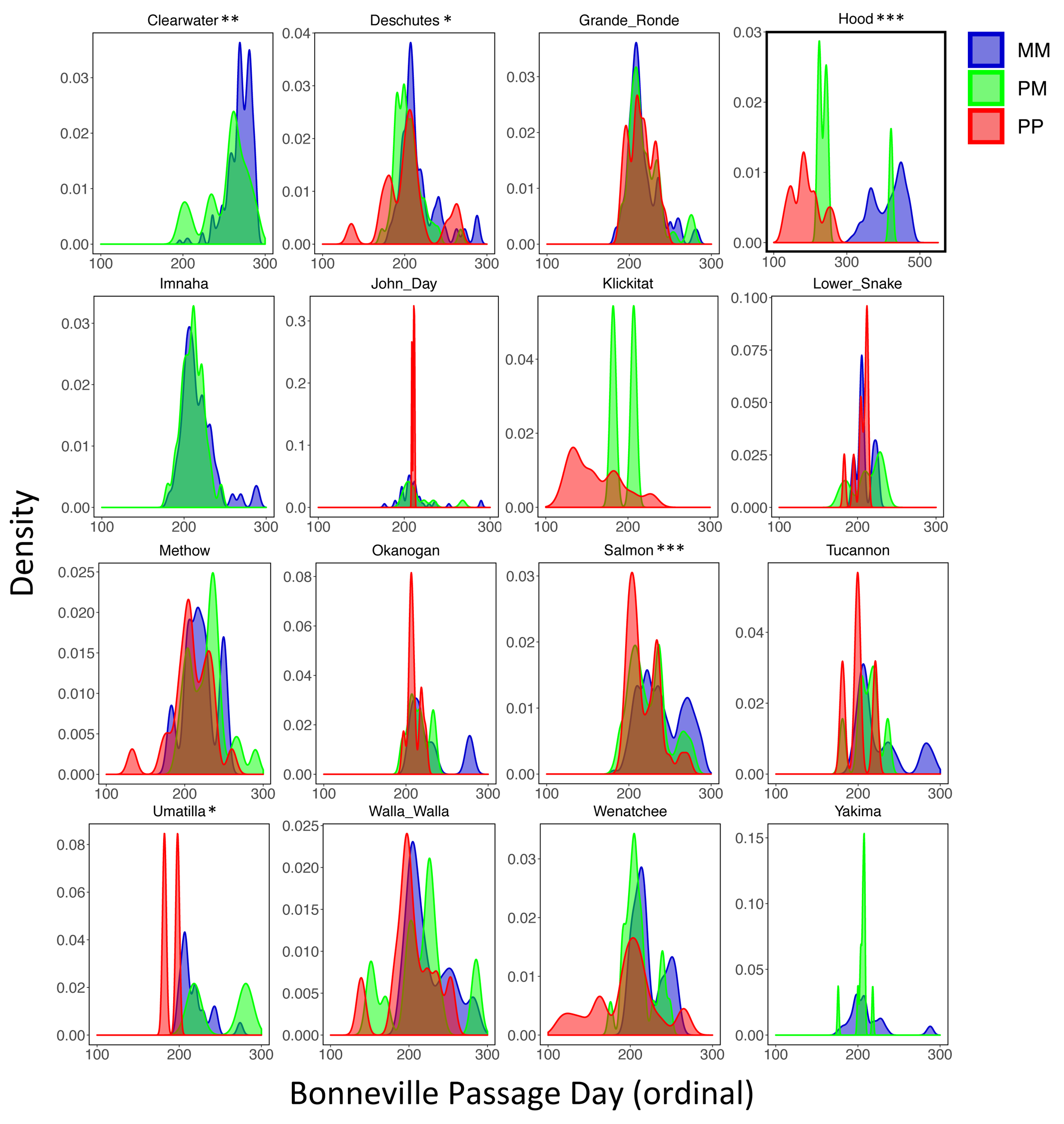

Supplement: Supplementary file 7 — Fig S7 [file EVA-13-2836-s007.tif]

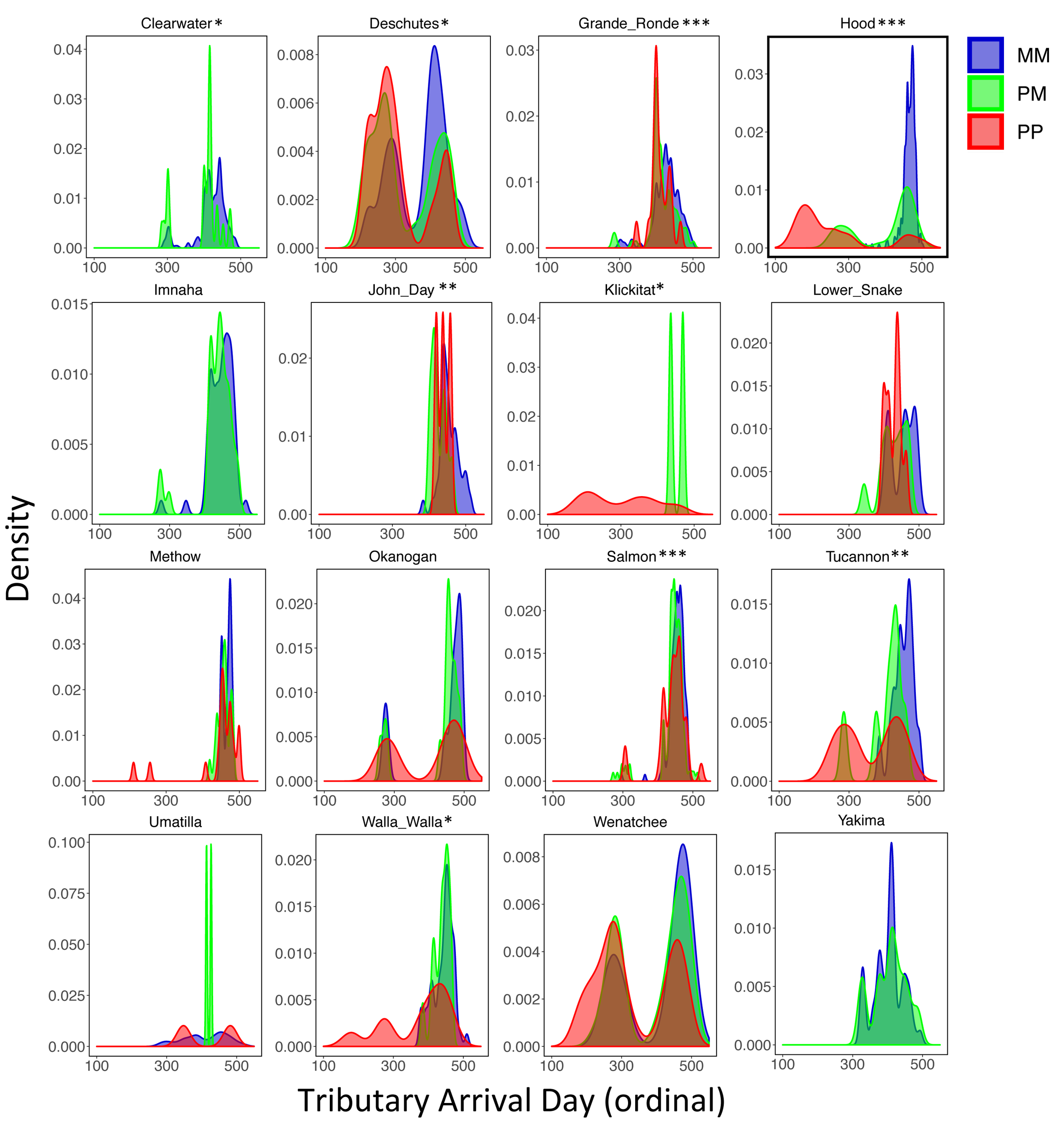

Supplement: Supplementary file 8 — Fig S8 [file EVA-13-2836-s008.tif]

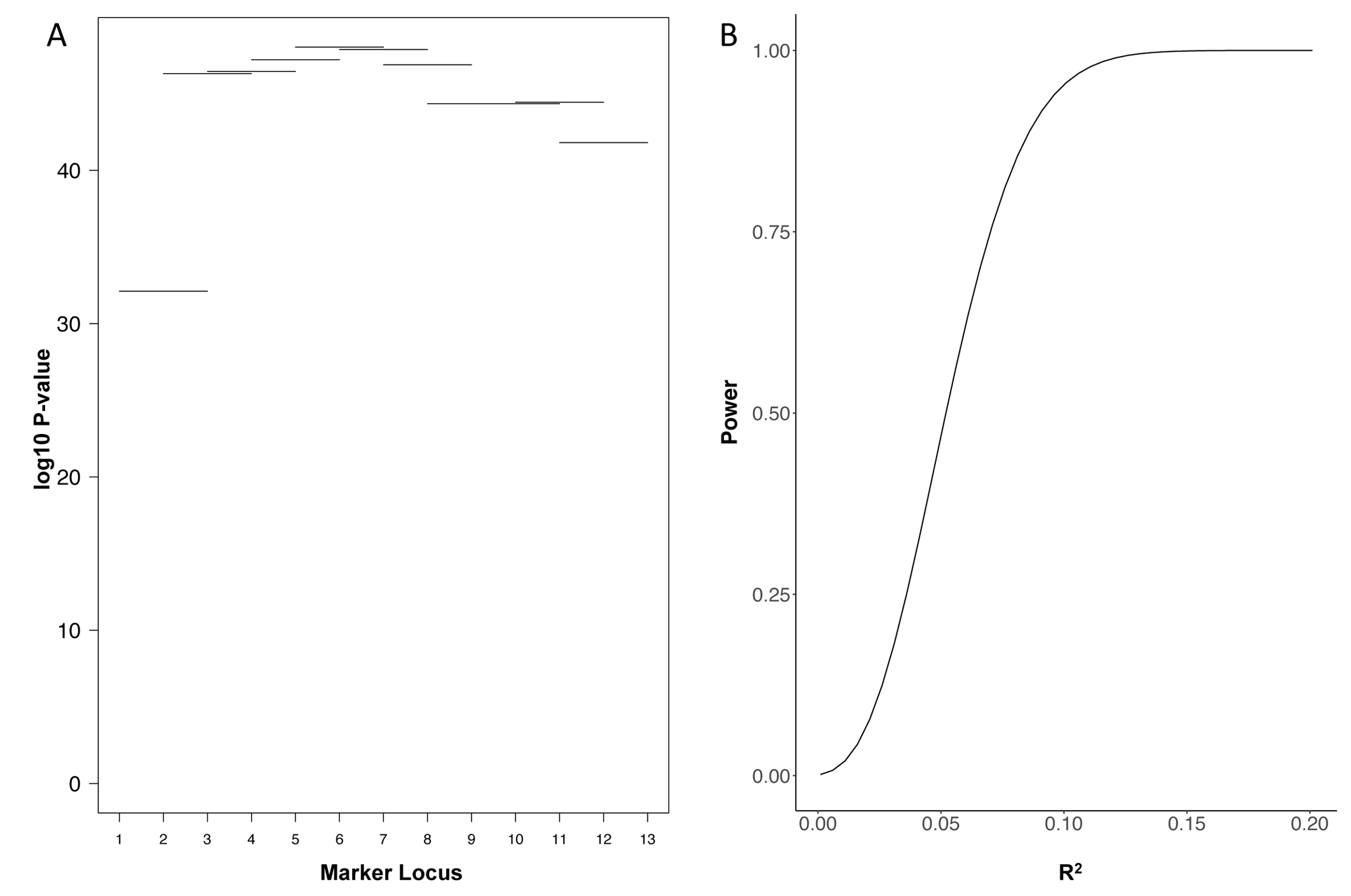

Supplement: Supplementary file 9 — Fig S9 [file EVA-13-2836-s009.tif]

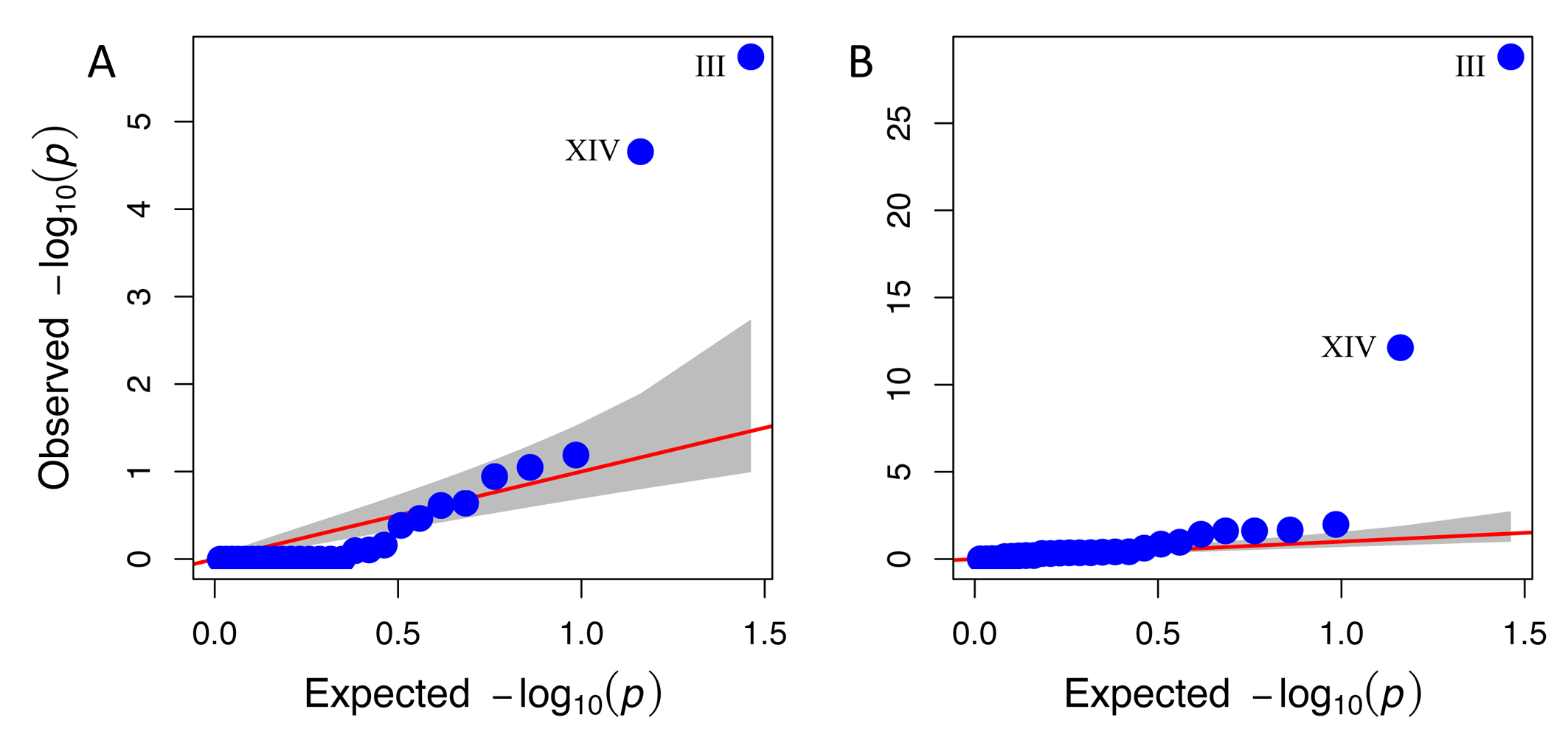

Supplement: Supplementary file 10 — Fig S10 [file EVA-13-2836-s010.tif]

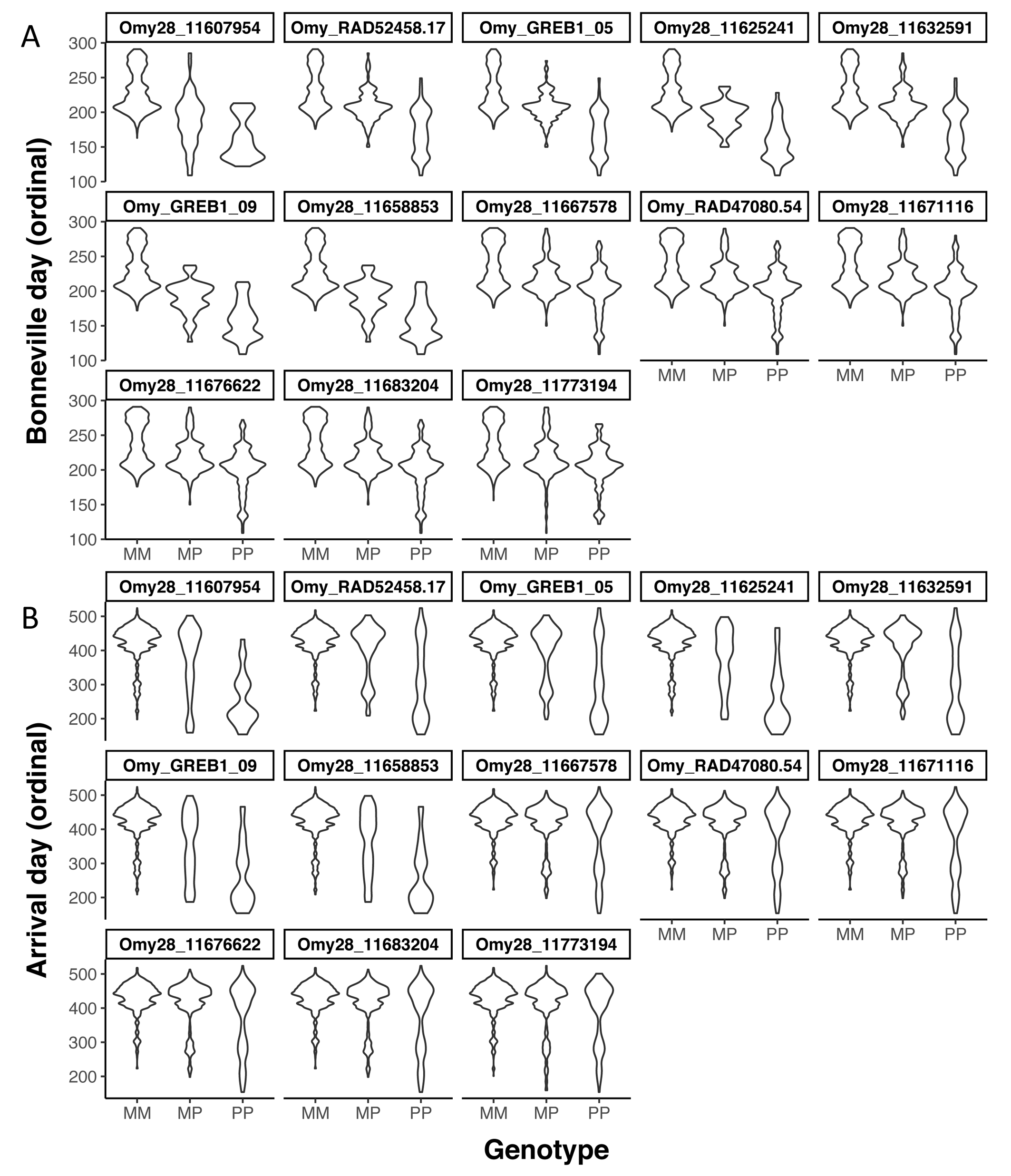

Supplement: Supplementary file 11 — Fig S11 [file EVA-13-2836-s011.tif]

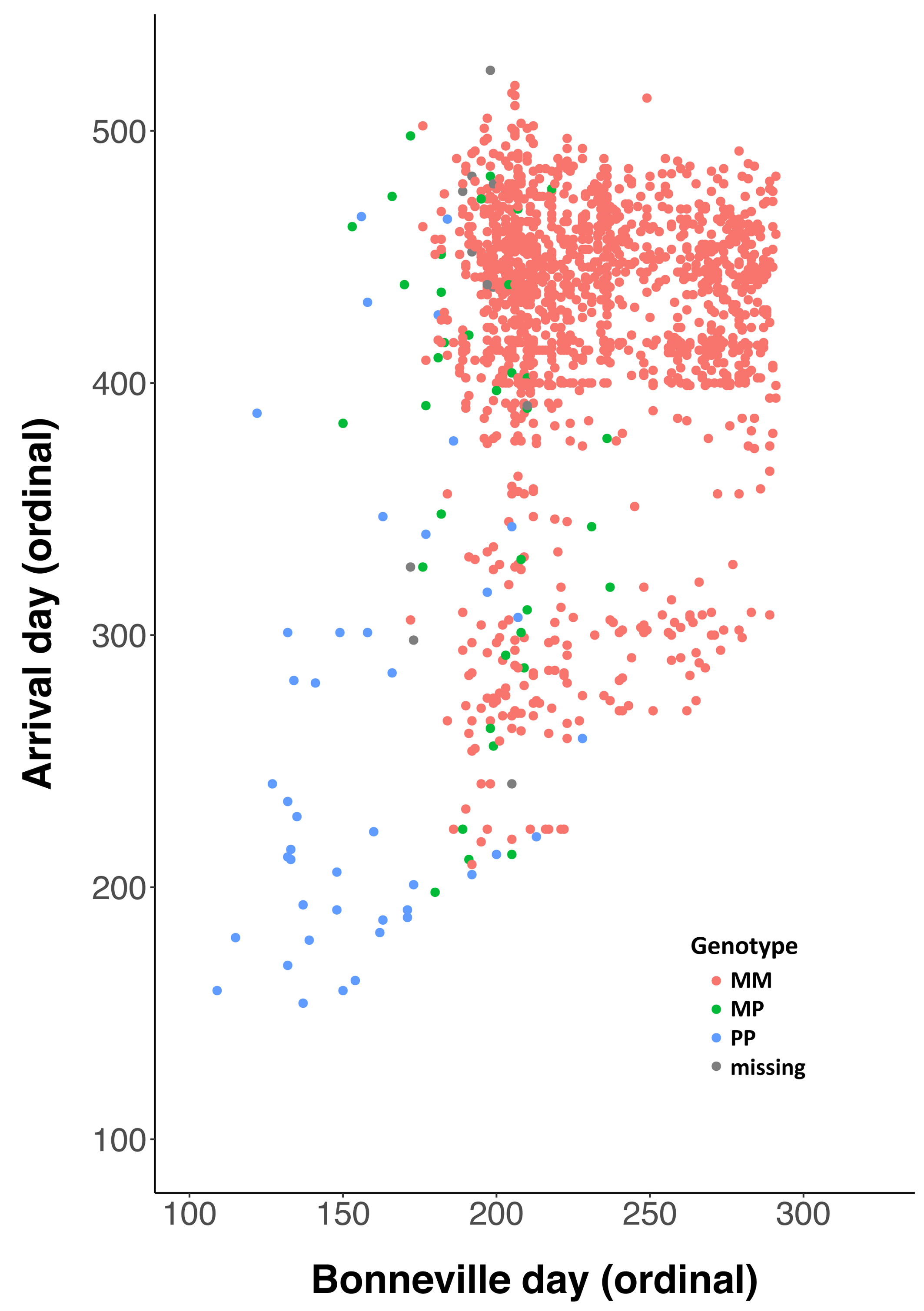

Supplement: Supplementary file 12 — Fig S12 [file EVA-13-2836-s012.tif]

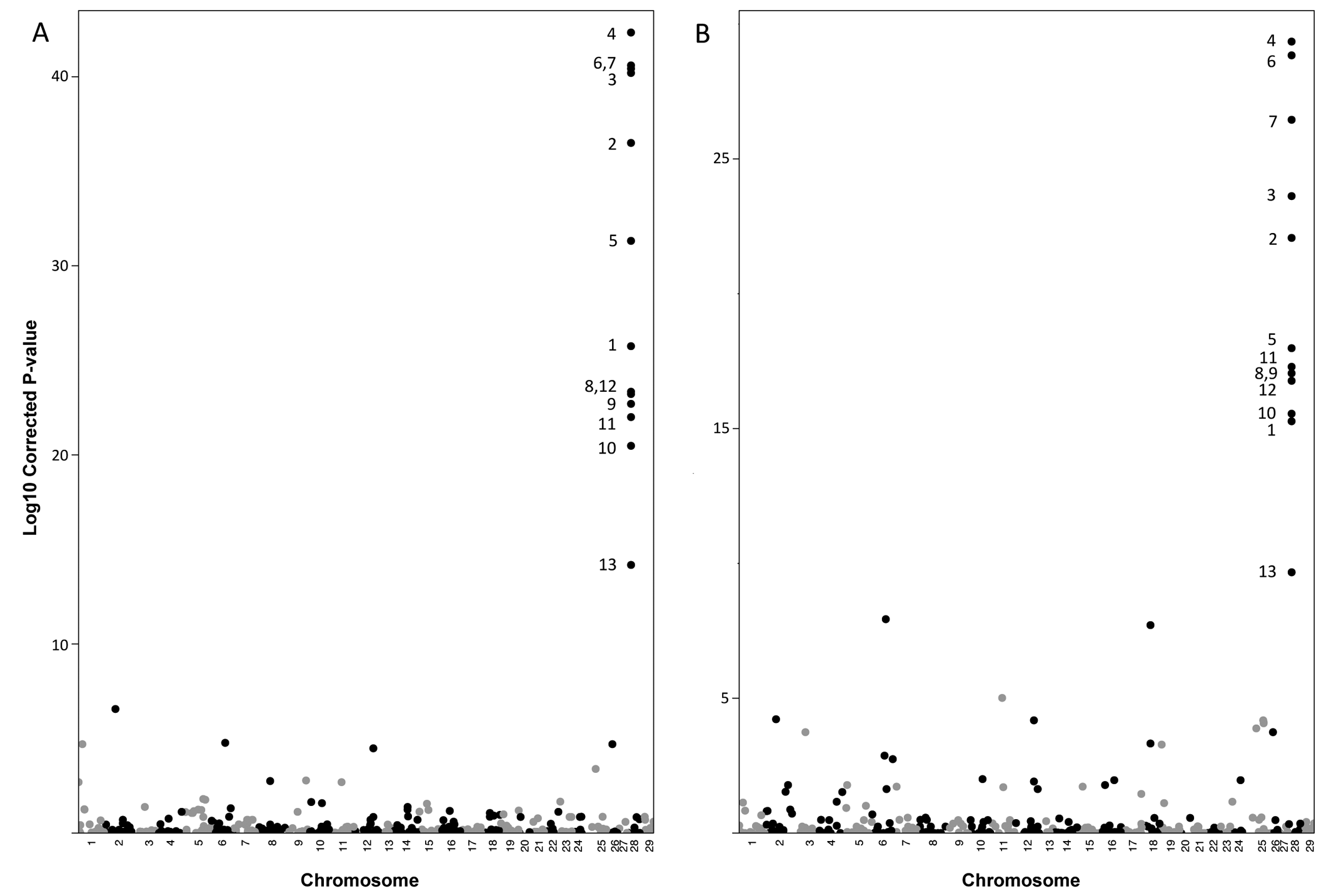

Supplement: Supplementary file 13 — Fig S13 [file EVA-13-2836-s013.tif]

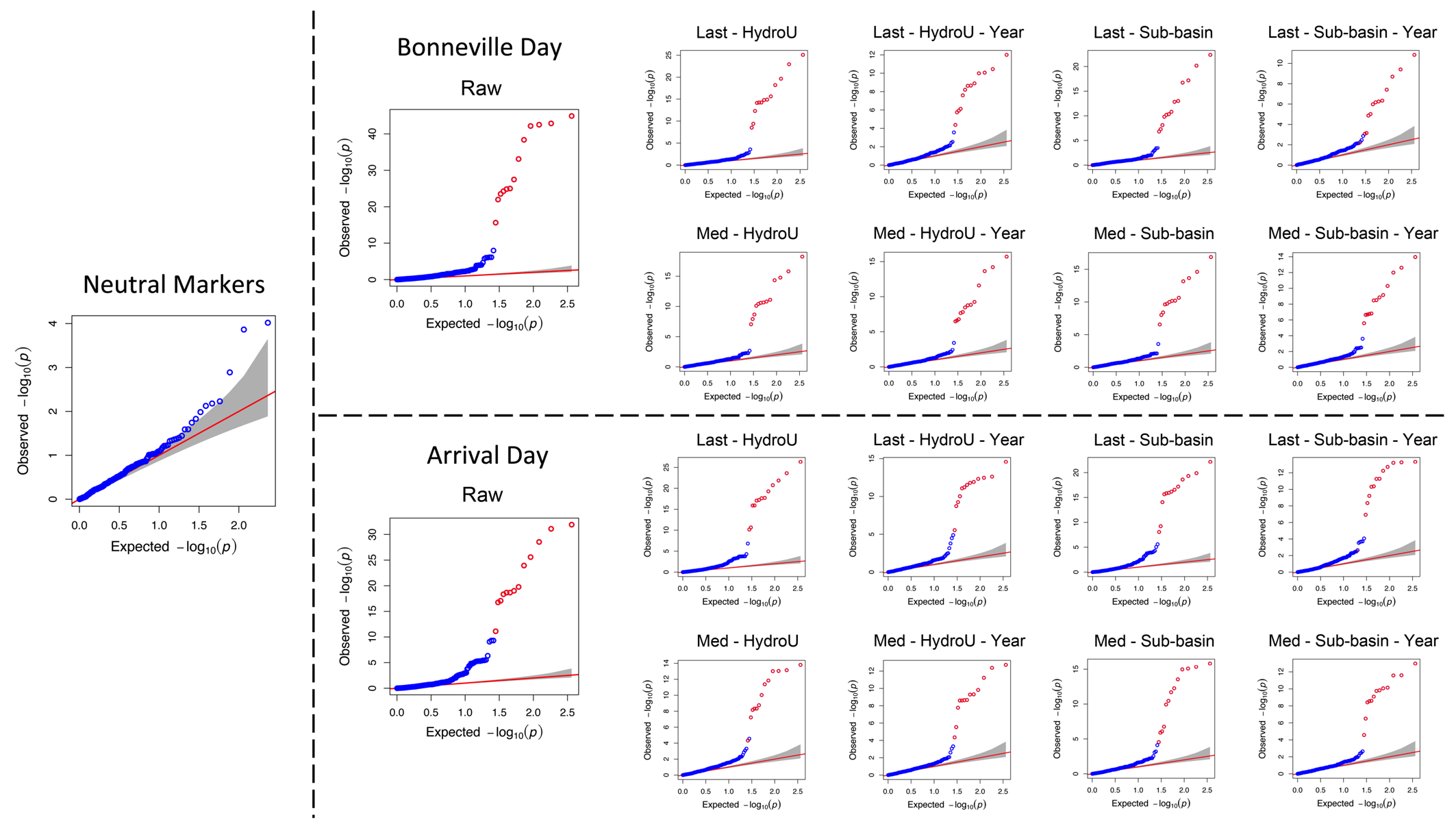

Supplement: Supplementary file 14 — Fig S14 [file EVA-13-2836-s014.tif]

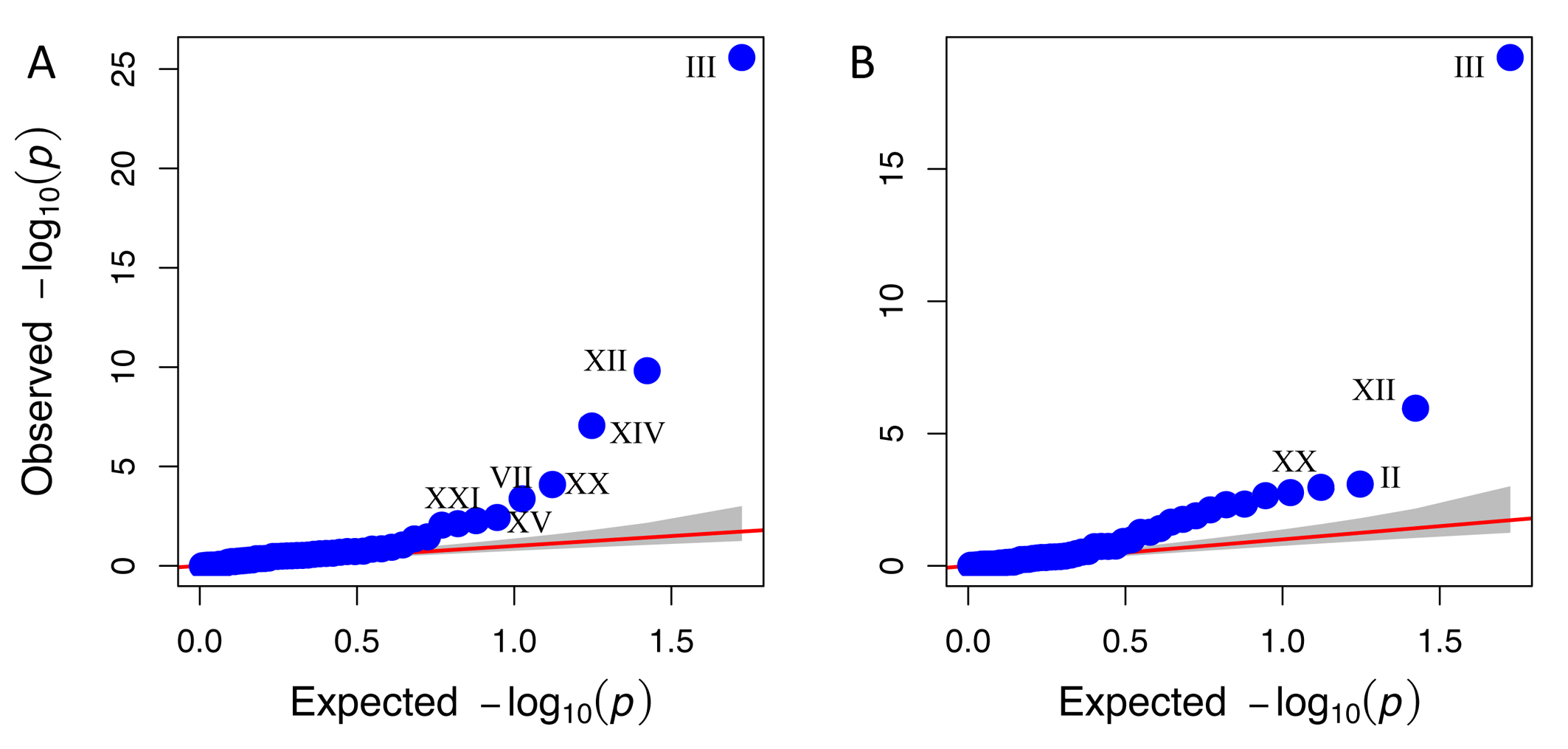

Supplement: Supplementary file 15 — Fig S15 [file EVA-13-2836-s015.tif]

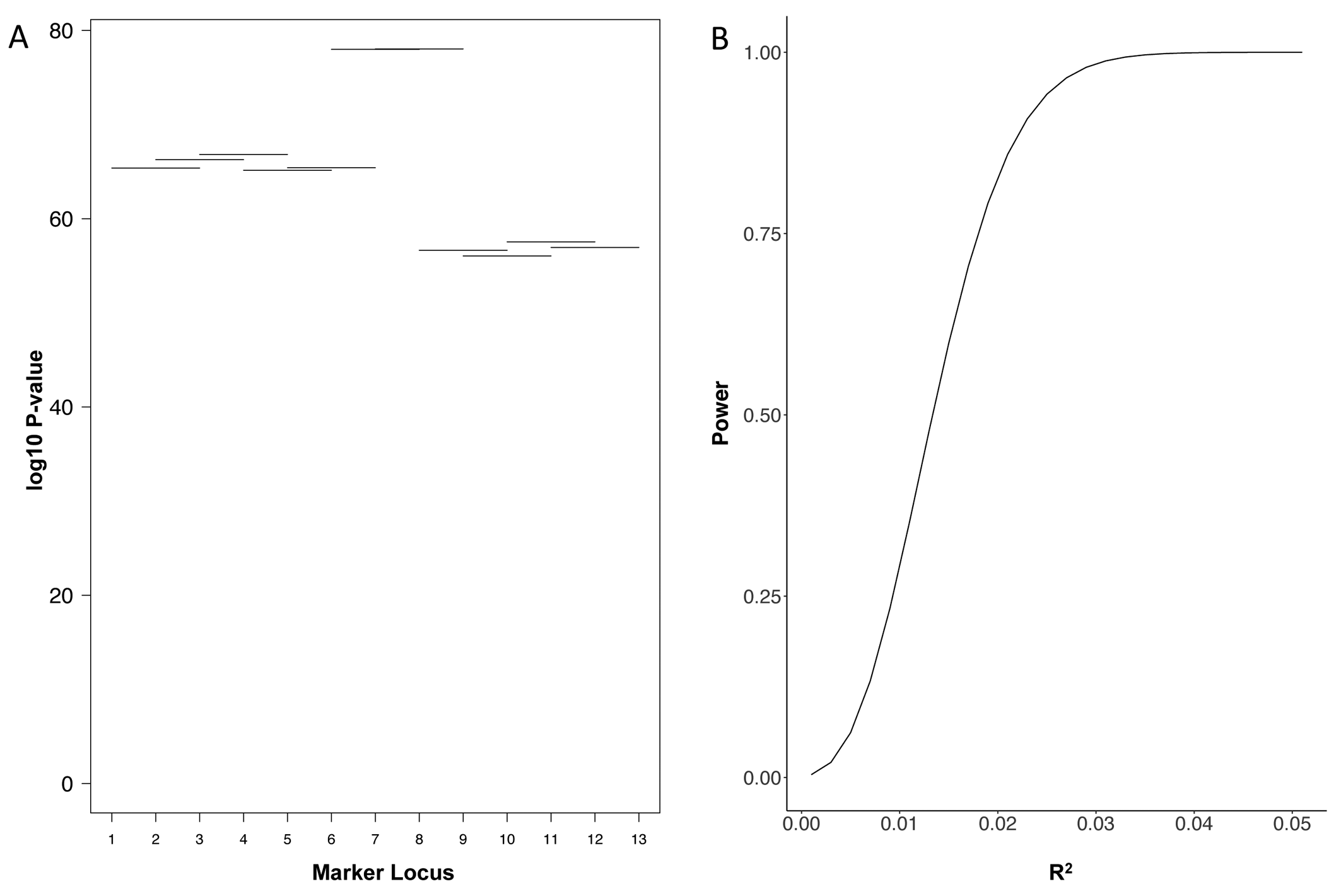

Supplement: Supplementary file 16 — Fig S16 [file EVA-13-2836-s016.tif]

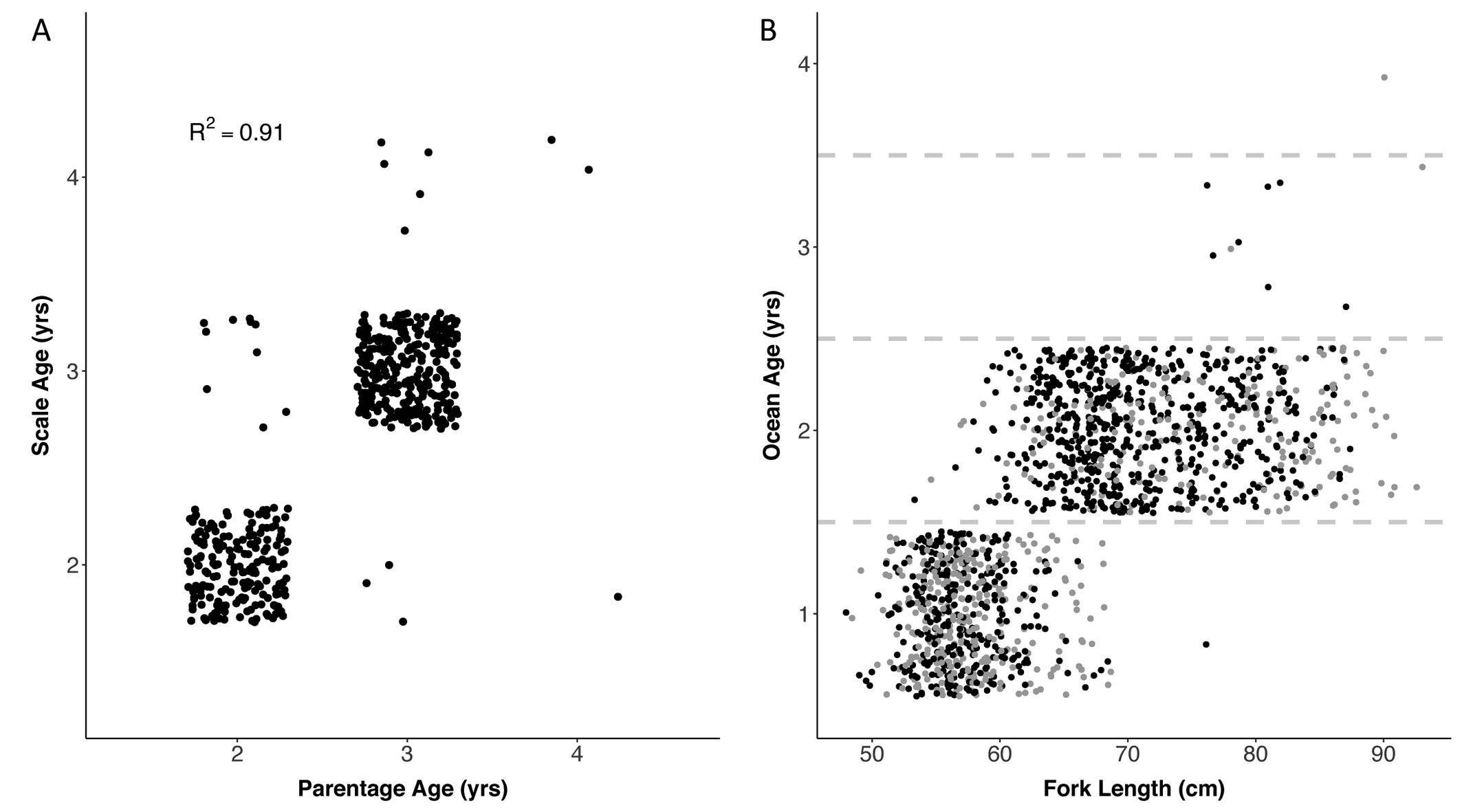

Supplement: Supplementary file 17 — Fig S17 [file EVA-13-2836-s017.tif]

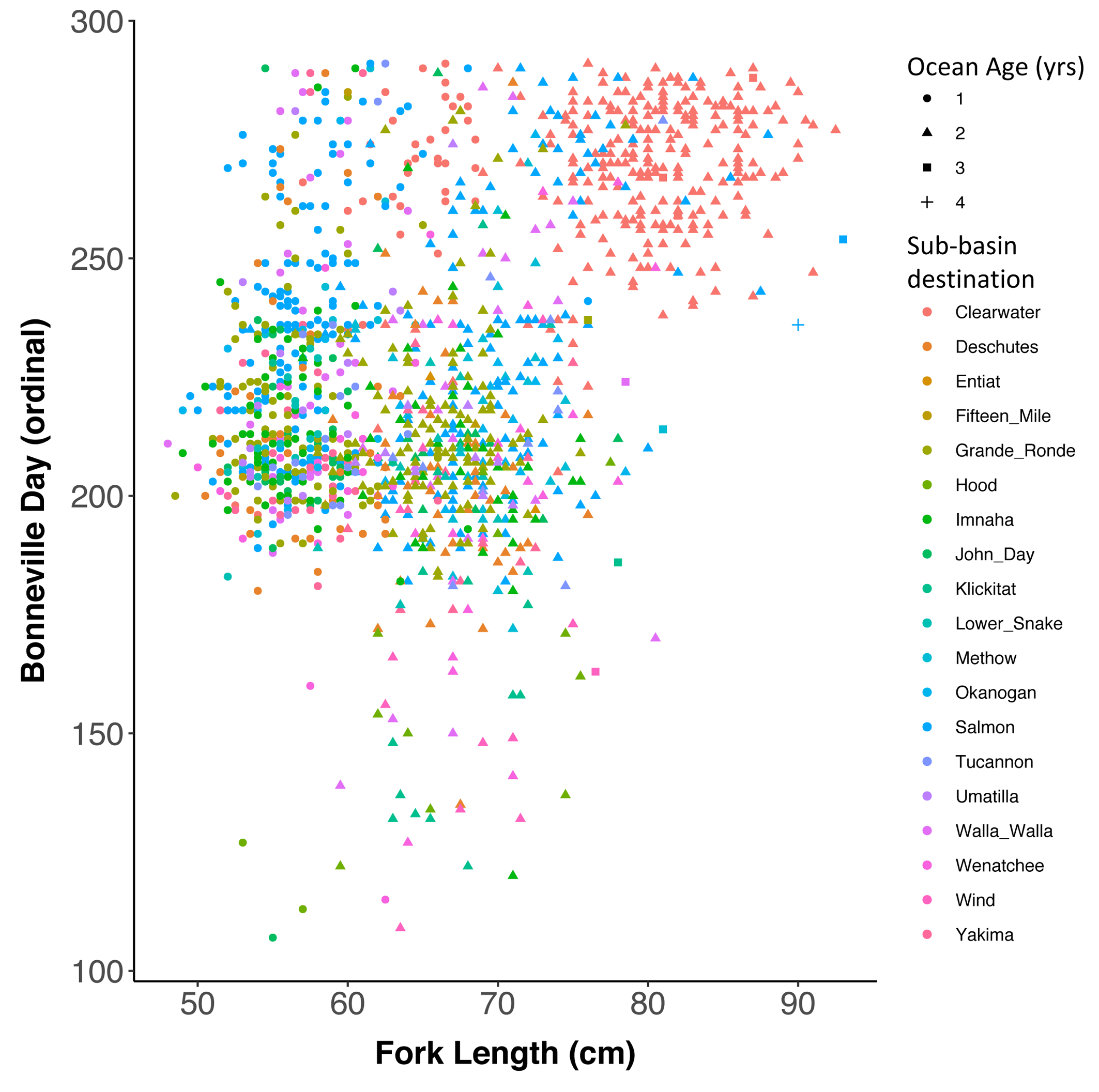

Supplement: Supplementary file 18 — Fig S18 [file EVA-13-2836-s018.tif]

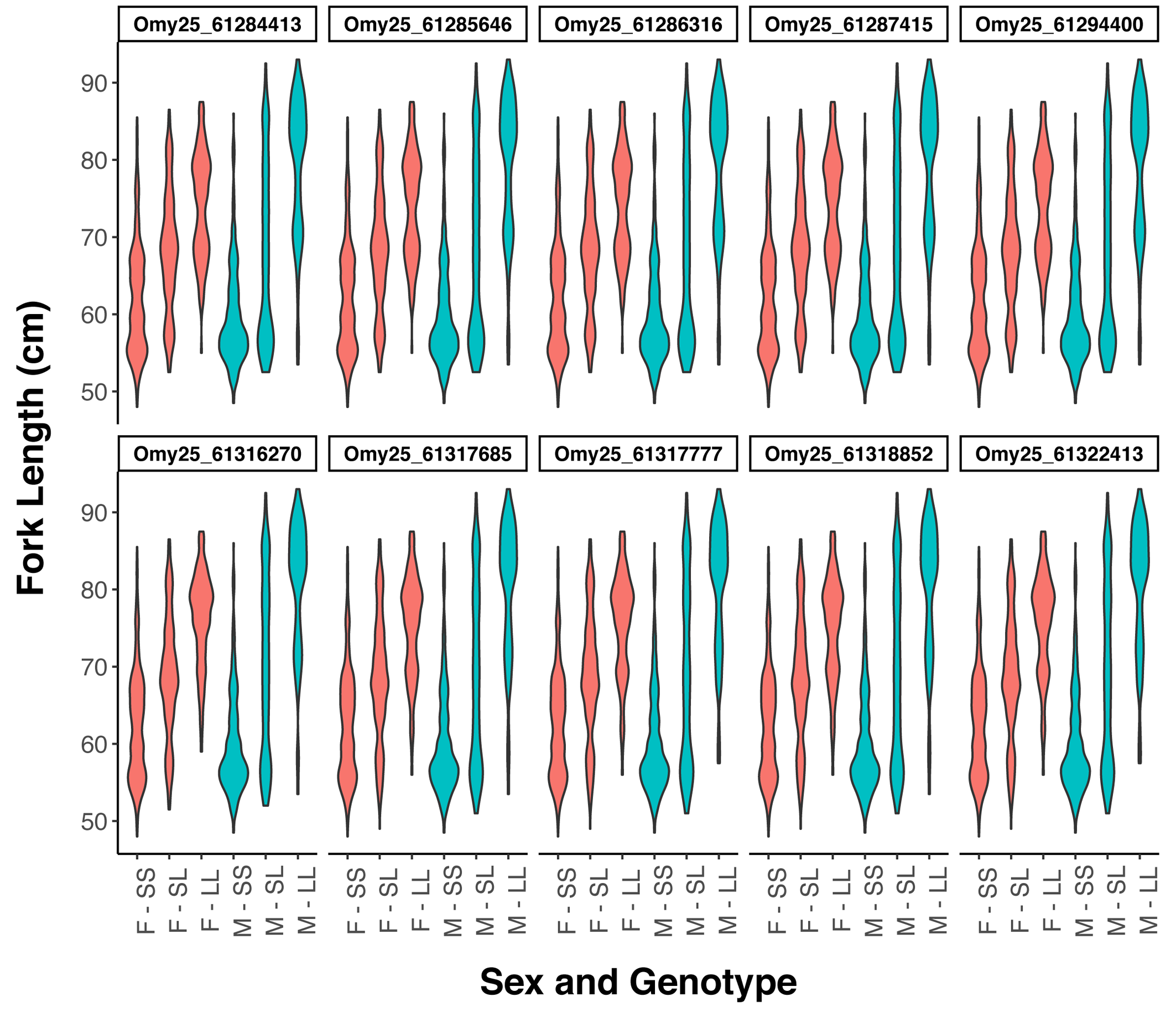

Supplement: Supplementary file 19 — Fig S19 [file EVA-13-2836-s019.tif]

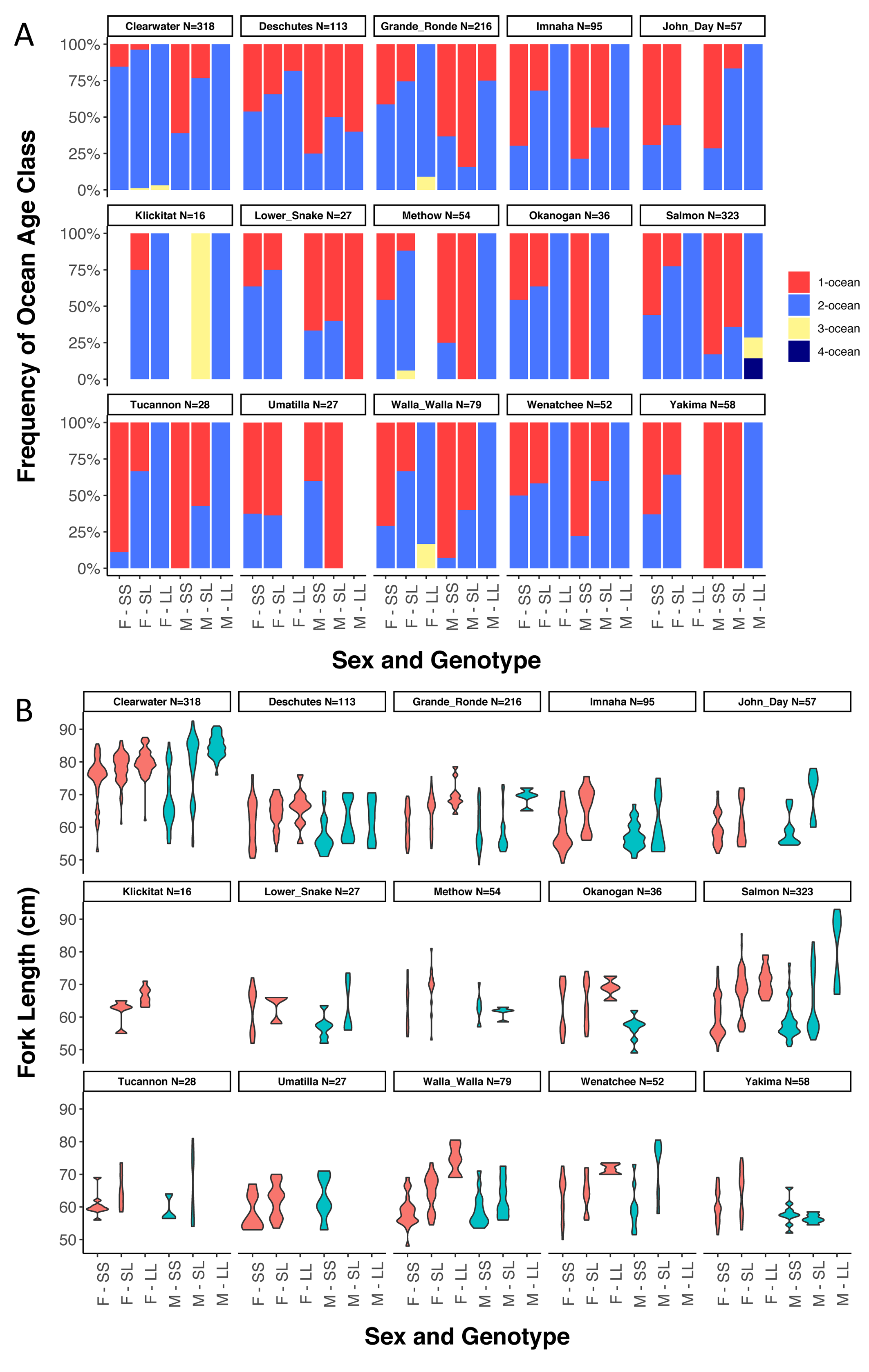

Supplement: Supplementary file 20 — Fig S20 [file EVA-13-2836-s020.tif]

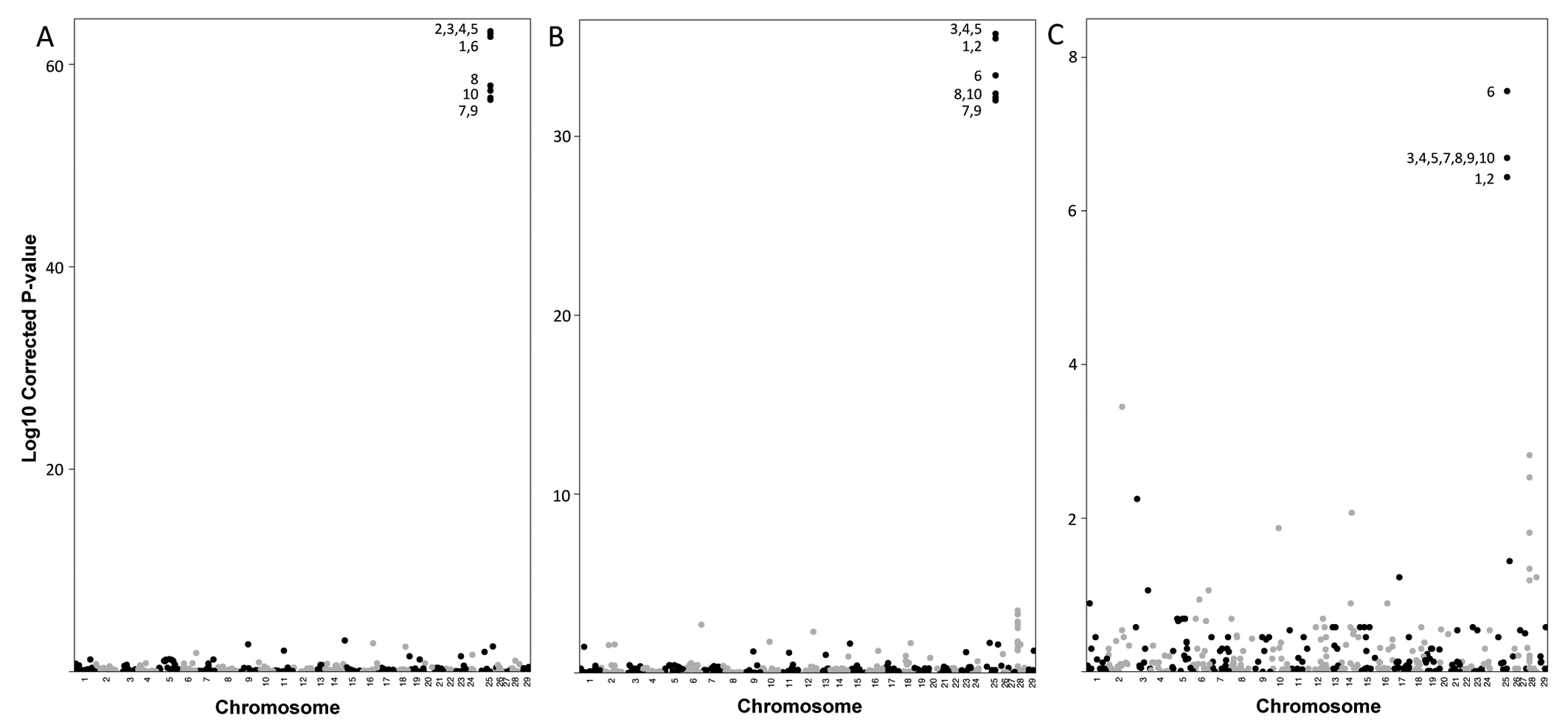

Supplement: Supplementary file 21 — Fig S21 [file EVA-13-2836-s021.tif]

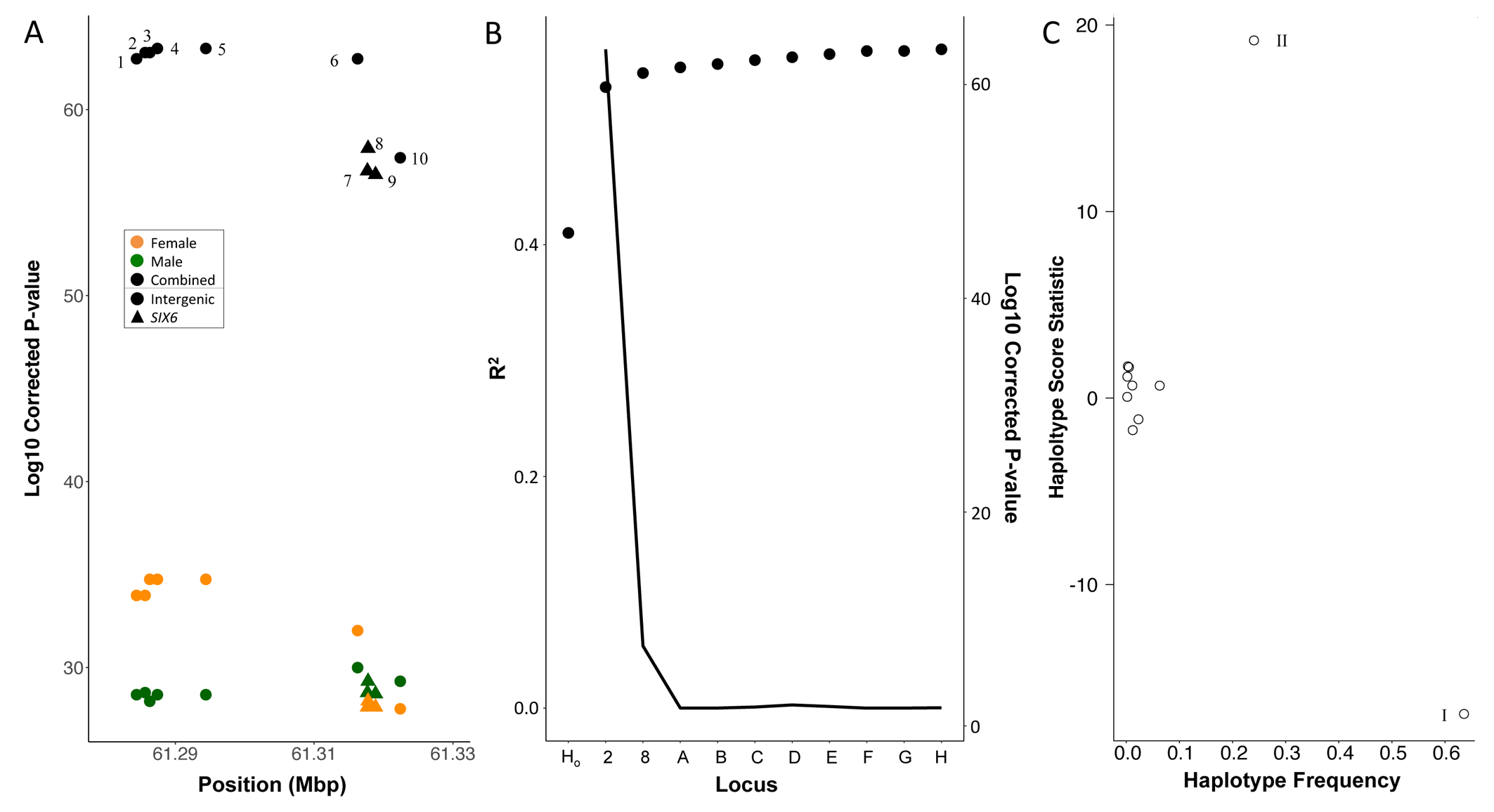

Supplement: Supplementary file 22 — Fig S22 [file EVA-13-2836-s022.tif]

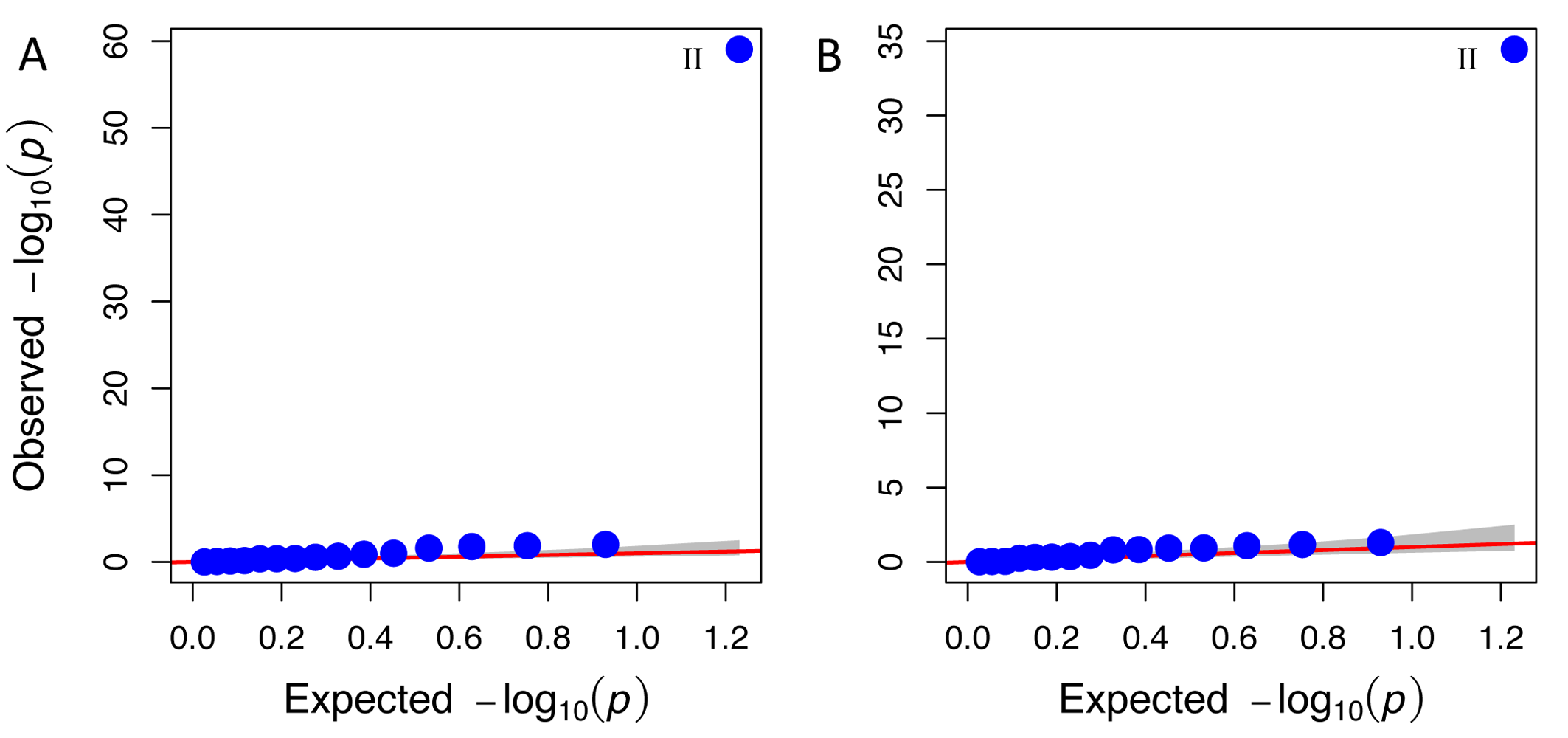

Supplement: Supplementary file 23 — Fig S23 [file EVA-13-2836-s023.tif]

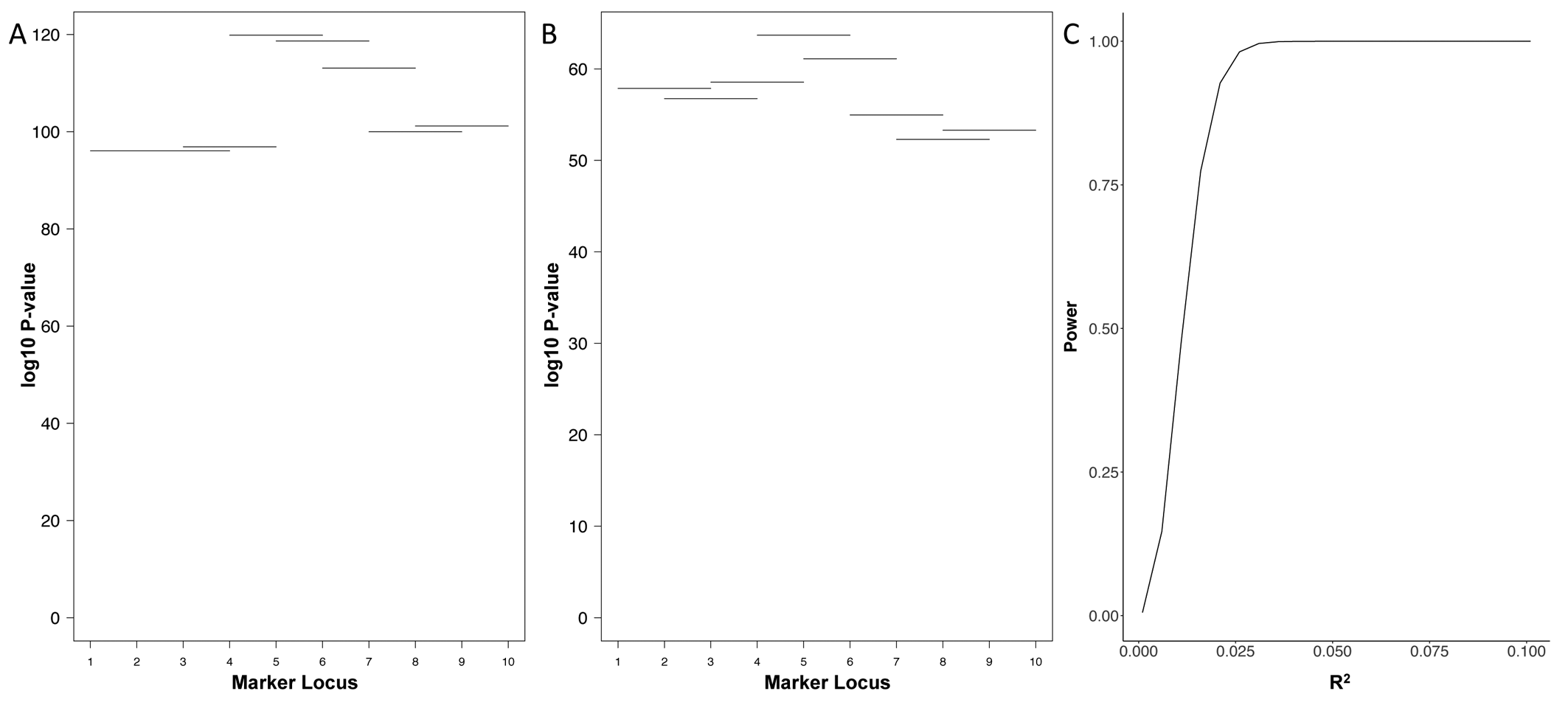

Supplement: Supplementary file 24 — Fig S24 [file EVA-13-2836-s024.tif]
